# Cranial biomechanics underpins high sauropod diversity in resource-poor environments

**DOI:** 10.1098/rspb.2014.2114

**Published:** 2014-11-22

**Authors:** David J. Button, Emily J. Rayfield, Paul M. Barrett

**Affiliations:** 1 School of Earth Sciences, University of Bristol, Life Sciences Building, 24 Tyndall Avenue, Bristol BS8 1TP, UK; 2 Department of Earth Sciences, The Natural History Museum, Cromwell Road, London SW7 5DB, UK

**Keywords:** niche partitioning, megaherbivores, sauropod, Morrison Formation, biomechanics, finite-element analysis

## Abstract

High megaherbivore species richness is documented in both fossil and contemporary ecosystems despite their high individual energy requirements. An extreme example of this is the Late Jurassic Morrison Formation, which was dominated by sauropod dinosaurs, the largest known terrestrial vertebrates. High sauropod diversity within the resource-limited Morrison is paradoxical, but might be explicable through sophisticated resource partitioning. This hypothesis was tested through finite-element analysis of the crania of the Morrison taxa *Camarasaurus* and *Diplodocus*. Results demonstrate divergent specialization, with *Camarasaurus* capable of exerting and accommodating greater bite forces than *Diplodocus*, permitting consumption of harder food items. Analysis of craniodental biomechanical characters taken from 35 sauropod taxa demonstrates a functional dichotomy in terms of bite force, cranial robustness and occlusal relationships yielding two polyphyletic functional ‘grades’. Morrison taxa are widely distributed within and between these two morphotypes, reflecting distinctive foraging specializations that formed a biomechanical basis for niche partitioning between them. This partitioning, coupled with benefits associated with large body size, would have enabled the high sauropod diversities present in the Morrison Formation. Further, this provides insight into the mechanisms responsible for supporting the high diversities of large megaherbivores observed in other Mesozoic and Cenozoic communities, particularly those occurring in resource-limited environments.

## 1. Introduction

Large herbivores are primarily limited by their high gross energy requirements, and so ultimately by plant productivity [1,2]. Despite this, very high diversities of megaherbivores (those greater than 10^3^kg [3]) in extant and Neogene mammalian communities are recognized from limiting environments [4–6]. An extreme example of this paradox is presented by the fauna of the Late Jurassic Morrison Formation of North America. The Morrison contains a uniquely high abundance of megaherbivores [7], although it was an (at least seasonally) arid environment [8–10] of limited productivity [7]. The fauna is known for its rich dinosaur remains, including at least nine ornithischian and 12 theropod genera. [11]. However, in terms of biomass, this community was dominated by sauropods [11], including 10 named genera (*Amphicoelias*, *Apatosaurus*, *Barosaurus*, *Brachiosaurus*, *Camarasaurus*, *Diplodocus*, *Haplocanthosaurus*, *Kaateodocus*, *Supersaurus* and *Suuwassea*) and several unnamed taxa [11,12]. Although the Morrison Formation extends for 6 000 000 km^2^ and was deposited over a period of 10 Myr [11,13] as many as three to five genera are found together at individual localities [11,12], indicating taxon co-occurrence. Sauropods were characterized by very large body size, with Morrison taxa ranging from 7 to 47 tonnes [11]. Given that sauropods included the largest terrestrial vertebrates of all time, reaching masses in excess of 80 tonnes [14], they illuminate the physical upper limits acting upon terrestrial life [15,16] and would have exerted powerful ecological impacts [7]. Herbivore populations may be controlled by resource availability, interspecific competition for resources and/or predator activity [17]. Although predation pressure would have been significant on juveniles, the large size of adult sauropods would have rendered them relatively immune to predation [7,15]. As a result of this, the limited productivity of the Morrison Formation environment and their large individual energy requirements, competition for resources would probably have been the ultimate limiting factor acting upon Morrison sauropod communities [7].

High species richness in extant resource-limited herbivore communities is facilitated by dietary niche partitioning [18–24]. Dietary and foraging specializations may be preserved in skeletal and dental correlates of feeding ecology [25–27], such as the correlation observed between muzzle shape and incisor width with feeding height and dietary selectivity in extant ungulates [25,28]. These correlates, together with tooth meso- and microwear data [27], enable formulation and testing of niche partitioning hypotheses between extinct taxa [29–31].

Dietary niche partitioning has been proposed as important in sauropod-dominated communities based on the high levels of disparity in their craniodental anatomy [32–34]. This disparity can be expressed as a spectrum between two morphotypes [35]. The plesiomorphic ‘broad-crowned’ condition features shearing dentitions in robust skulls. The derived diplodocoid and titanosaur lineages show the independent derivation of a ‘narrow-crowned’ morphology of a reduced dentition of peg-like teeth occupying a gracile skull with a narrow, inclined adductor chamber. End members of this spectrum are exemplified by *Camarasaurus* and *Diplodocus*, respectively, two of the most abundant and regularly co-occurring sauropod taxa in the Morrison Formation [11]. This dichotomy has led to *Camarasaurus* being hypothesized as adapted towards greater bite forces, the procurement of coarser fodder and potentially engaging in greater oral processing, while *Diplodocus* has been characterized as more limited in bite force and dietary choice [32–34], potentially carrying out specialized ‘branch-stripping’ behaviours, where movements of the neck would have been used to rake the dentition through plant material [34,36]. However, the functional relevance of these morphotypes and their significance for ecological partitioning has not been tested within a biomechanical framework.

Here we address this problem through two complementary analyses. First, we used myological reconstruction and detailed three-dimensional finite-element analysis (FEA). This is a modelling technique that calculates stress and strain in a structure in response to an applied load. Almost all FEA work in palaeontology has been concerned with investigation of individual taxa, whereas comparative studies within an evolutionary context have been rare [37,38]. Second, we used biomechanically relevant measures to quantify the disparity of sauropod craniodental systems. We applied three-dimensional FEA to the sauropod taxa *Camarasaurus* and *Diplodocus*, and estimated sauropod functional disparity across the clade as a whole in order to test evolutionary and ecological hypotheses in a rigorous and comparative biomechanical context.

## 2. Material and methods

### (a) Virtual skull and muscle reconstruction

The skull and mandible of CMNH 11338, a juvenile *Camarasaurus lentus*, and the skull and a mandible cast of CMNH 11161, an adult *Diplodocus carnegii*, were CT scanned at the O'Bleness Memorial Hospital, Ohio, by L. M. Witmer (who made the scans available for this study). Scan data were imported into Avizo (v. 6.3.1 and 7, FEI Visualization Science Group). A complete skull reconstruction of *Camarasaurus* was produced by labelling each element separately in the Avizo segmentation editor, with warping, translocation and mirroring of elements (using a custom script written by S. Lautenschlager) used to correct for deformation and restore missing elements. The low ontogenetic variability in the skull morphology of *Camarasaurus* [39] meant that the scanned skull could then be scaled by 180% to match the proportions of an adult *C. lentus* (based upon DINO 28 [40]) to permit quantitative comparison between adult *Camarasaurus* and *Diplodocus*. The total skull surface area was measured for the adult size-scaled CMNH 11338 and the restored CMNH 11161 skull as used by Young *et al*. [36] using the Avizo material statistics module. The jaw musculature of both taxa was digitally reconstructed from the skull models following the methodology laid out by Lautenschlager [41]. Muscle origination and insertion areas were identified on the basis of osteological correlates [42]. Total muscle volumes were deduced according to spatial constraints and topological relations of the muscles and other soft tissues [41–43], and by comparison with pre-existing muscle reconstructions of *Diplodocus* [42,44]. Muscle forces were calculated using the ‘dry skull method’ [45]. Individual muscle volumes were measured in Avizo, and physiological cross-sectional area was calculated by dividing the volume by the total length of the muscle (see electronic supplementary material, §8). Total length is only an approximation of total fibre length as it does not take into account muscle pennation, but was used here to minimize *ad hoc* assumptions. Contractile force was calculated by multiplying this area by a specific tension value reported from vertebrate muscle, 392 kPa [46] (for sensitivity analyses employing a range of values, see electronic supplementary material, §9). Craniocervical muscle force was calculated in a similar way, with cross-sectional areas calculated from occipital insertion areas [47–50], and estimated from lateral and anterior views of the vertebrae after [51].

Muscle abbreviations used are as follows. Jaw adductors: m. AMEP, m. adductor mandibulae externus profundus; m. AMEM, m. adductor mandibulae externus medialis; m. AMES, m. adductor mandibulae externus superficialis; m. AMP, m. adductor mandibulae profundus; m. PSTs, m. pseudotemporalis superficilias; m. PTd, m. pterygoideus dorsalis; m. PTv, m. pterygoideus ventralis. Craniocervical musculature (nomenclature as in [47]): m. c., m. complexus; m. i.c., m. iliocastalis capitis; m. l.c.p., m. longissimus capitis profundus; m. l.c.s., m. longissimus capitis superficialis; m. r.c.v., m. rectis capitis ventralis; m. s.c., m. splenius capitis; m. t.c., m. transversospinalis capitus.

### (b) Finite-element models

The completed skull model of *Camarasaurus* was imported into Hypermesh (v. 11, Altair), where the surface was ‘cleaned’ of errors to produce a higher-quality mesh (as tested using internal element checks in Hypermesh) of 877 796 tetrahedral elements and 194 844 nodes. Convergence testing [52] indicates that this is a sufficient number of elements to describe stress and strain patterns observed in the skull (see electronic supplementary material, §9). The skull was loaded with the calculated muscle forces using a custom-built macro supplied by Altair UK, which loads multiple nodes across the muscle origination site along a vector projected towards a node representing the insertion site on the mandible. Material properties of vertebrate enamel (Young's modulus = 80 GPa, Poisson's ratio = 0.3 [53]), dentine (Young's modulus = 21 GPa, Poisson's ratio = 0.31 [54]) and bovine Haversian bone (Young's modulus = 23.1 GPa, Poisson's ratio = 0.29 [55]) were applied as appropriate (see electronic supplementary material, §9). The completed model was then solved in Abaqus (v. 6.10.2, Dassault Systèmes Simulia). The *Diplodocus* model of Young *et al*. [36] was modified in Hypermesh with the updated jaw adductor muscle forces calculated herein and the addition of loads from the craniocervical musculature.

Constraints were applied to the quadrates, preventing translation in the *x-*, *y-* and *z*-axes, and in the biting teeth, constraining against translation in the *y*-axis (the axis of biting). The four anterior-most teeth were constrained in both taxa, replicating an anterior bite. For each constraint point a distributing coupling constraint (DCC) was applied in Hypermesh. A DCC comprises a series of rigid links that spread the constraint over multiple nodes, reducing problems of unrealistically high stresses that can result from point constraints [56] (see electronic supplementary material, §9).

To obtain bite forces from the models, the tooth constraints were altered, with a single node on each biting tooth fully constrained to produce a reaction force [57]. Anterior bite force was taken as the sum of the reaction force from two point constraints, one on the left and one on the right anterior-most teeth. Posterior bite force was taken as the sum of reaction forces from two point constraints between the left and right posterior-most teeth.

### (c) Finite-element analyses

To compare both *Camarasaurus* and *Diplodocus* two groups of analyses were performed. In each, comparison of von Mises stress distribution and magnitude were made between *Camarasaurus* and *Diplodocus*. von Mises stress represents a single scalar approximating the ‘overall stress', and so the proximity to failure, from the combination of the three principal stresses at any point [58].

#### (i) Ecological comparison

The ecological comparison is intended to compare the relative performance of each animal as ecological competitors. For this comparison, the skull of *Diplodocus* and reconstructed muscle volumes were retained as actual (adult) size and compared with the skull and muscle volumes of *Camarasaurus* scaled to adult size (see above and electronic supplementary material, §8).

#### (ii) Structural comparison

The structural comparison is intended to test the relative performance of the skulls of each taxon purely in the context of shape differences. This necessitates standardization to remove the effects of size and differing muscle loads [59]. As the metric reported here is stress, the applied muscle force was scaled so that the ratio of total applied muscle force to skull surface area was equal for both taxa [59].

Analyses replicating ‘branch-stripping’ behaviour were also performed, including loading from the jaw musculature and plant-stripping forces following Young *et al*. [36], and also including the loading consequences of the craniocervical musculature. For these additional analyses see electronic supplementary material, §§8 and 11.

### (d) Biomechanical functionspace analysis

Twenty craniodental functional characters were measured in 35 sauropod species (see electronic supplementary material, §§1–3). Taxa were grouped into a basal ‘broad-crowned’ evolutionary grade, diplodocoids and titanosauriformes, with the last two split more finely into the Rebbachisauridae, Dicraeosauridae, Diplodocidae, Brachiosauridae, *Euhelopus* and Titanosauria. Average measures were taken for taxa known from multiple well-preserved skulls. The functional characters include 17 biomechanically significant continuous metrics that together can be used to infer the functional properties of the skull and mandible (see electronic supplementary material, §3 for character descriptions). The remaining four characters represent binary tooth characters (a similar combination of continuous and binary characters was also used by Anderson *et al*. [60]). The continuous characters were standardized using the *z-*transformation. These transformed data were analysed using principal coordinate analysis (PCO), performed in PAST [61], to produce a multivariate biomechanical ‘functionspace’. PCO was used as it does not require a complete matrix; completeness of the biomechanical matrix was 75.3%. Differences in functionspace occupation between the groups listed above were tested using a non-parametric multivariate analysis of variance (npMANOVA) [62] using principal coordinate (PC) scores of the first 18 axes conducted in PAST (see electronic supplementary material, §6). The significance of the correlation of each of the characters with PC axes 1 and 2 was evaluated using the Spearman's rank order correlation coefficient (see electronic supplementary material, §3).

### (e) Biomechanical phylomorphospace

An informal supertree of the Sauropoda was constructed (see electronic supplementary material, §7 for details) and time-calibrated based on taxon occurrences dated to the stage level performed using the ‘timePaleophy’ function of the paleotree package [63] within R. The time-calibrated supertree was then mapped onto the first two PC axes of the biomechanical morphospace within R to yield the biomechanical phylomorphospace.

## 3. Results

### (a) Muscle reconstruction

**Figure 1.**
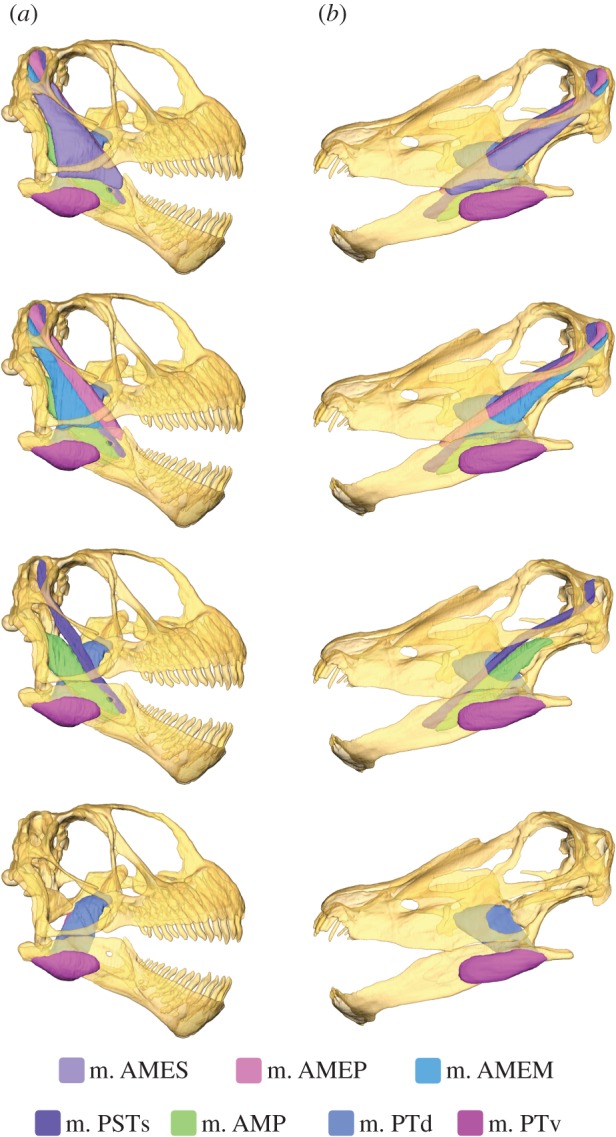
Model of the skull of (*a*) *Camarasaurus* and (*b*) *Diplodocus*, demonstrating the reconstructed jaw adductor musculature at four successive ‘depths'. See §2a for muscle abbreviations. For muscle forces, see table 1, and electronic supplementary material, table S6 and §S5.

Reconstruction of jaw musculature (figure 1 and table 1) demonstrates considerably larger muscle volumes for *Camarasaurus* than *Diplodocus. Camarasaurus* has a greater contribution from the m. adductor mandibulae externus than *Diplodocus* (38% versus 22%), with the palatal musculature more important in *Diplodocus*. Calculated bite forces are much greater for *Camarasaurus*, especially at the posterior-most bite point (table 1).

**Table 1.** Calculated jaw adductor muscle forces. Jaw adductor muscle forces calculated from reconstructed muscle volumes with bite forces resulting from finite-element models. These all represent maximum values; see electronic supplementary material, tables S5 and S6 for complete range in calculated values. See §2a for muscle abbreviations.

		*Camarasaurus* (N)	*Diplodocus* (N)
temporal muscles	m. AMES	592	175.22
	m. AMEP	227.4	40.77
	m. AMEM	312.4	95.65
	m. PSTs	154.8	103.1
palatal muscles	m. AMP	493.9	146.6
	m. PTd	611.5	407.7
	m. PTv	584.1	355.9
anterior bite force		981.8	234.5
posterior bite force		1859	324.2

**Figure 2.**
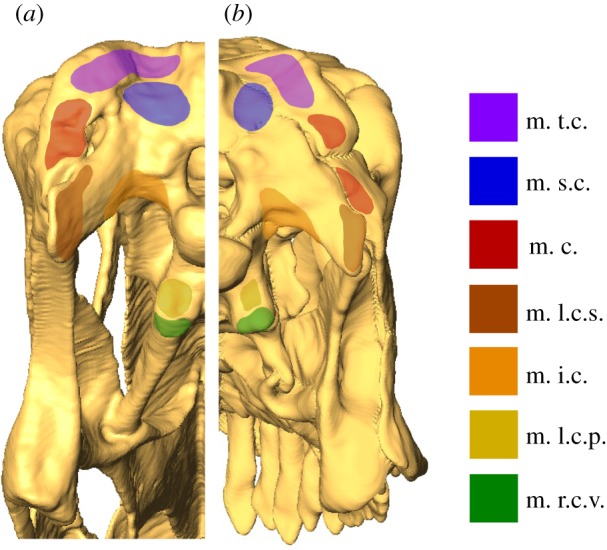
Posterior view of the skull models of (*a*) *Diplodocus* and (*b*) *Camarasaurus*, demonstrating the insertion areas of the craniocervical musculature for each. See §2a for muscle abbreviations. Skulls not to scale. For muscle forces, see table 2 and electronic supplementary material, §S8.

Reconstruction of craniocervical musculature insertion areas (figure 2 and table 2) demonstrates greater overall muscle volumes for *Camarasaurus*. However, if corrected for skull surface area the ventroflexors of *Diplodocus* are considerably more powerful than those of *Camarasaurus*. This is reflected in the relative contributions of the muscles, with the dorsiflexors more important in *Camarasaurus* versus greater importance of the ventroflexors in *Diplodocus*.

**Table 2.** Calculated craniocervical muscle forces. Maximum calculated forces of the craniocervical muscles of each taxon. For complete range of calculated values see electronic supplementary material, table S7. See §2a for muscle abbreviations.

		*Camarasaurus* (N)	*Diplodocus* (N)
dorsiflexors	m. s.c.	415.5	218.0
	m. t.c.	403.76	254.0
lateroflexors	m. c.	134.5	200.3
	m. l.c.s.	344.2	163.1
	m. i.c.	302.2	255.2
ventriflexors	m. l.c.p.	154.8	94.86
	m. r.c.v.	143.5	104.3

### (b) Finite-element analysis results

#### (i) Ecological comparison

**Figure 3.**
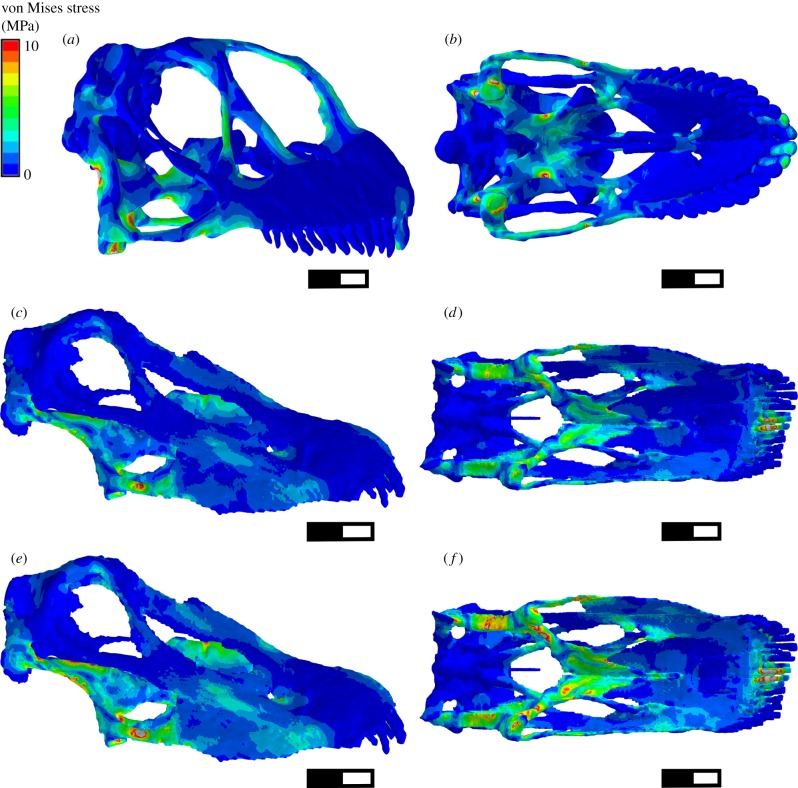
von Mises stress contour plots from FEA of *Camarasaurus* and *Diplodocus* under loading replicating static biting. (*a,b*) *Camarasaurus*, (*c,d*) *Diplodocus* (adult size; for ecological comparison see §2) (*e,f*) *Diplodocus* when scaled to equal applied load/surface area as *Camarasaurus* (for structural comparison see §2). Scale bar, 100 mm.

The finite-element model von Mises stress contour plots replicating muscle-driven static biting in adult-sized skulls for both taxa are similar (figure 3), with overall functionally induced stress low throughout the skull. Mean element stresses are slightly higher in *Diplodocus* (figure 3*a–d* and table 3). Maximum stress in *Camarasaurus* occurs in a localized area of the quadrate shaft (figure 3*a,b*). Elevated stresses are also located in the pterygoids, the biting teeth and in the thin bony bars of the skull. Maximum stresses observed in *Diplodocus* are higher than those seen in *Camarasaurus*. The very thin post-orbital, lacrimal and facial bones of *Diplodocus* experience only very low stress; instead elevated stresses are more concentrated within the palate, which is elongated and expanded in *Diplodocus*, as compared with *Camarasaurus*, through the dorsoposterior rotation of the posterior region of the skull.

**Table 3.** Model element stress comparison. Minimum, maximum and mean element von Mises stresses for each of the three different models. The ‘ecological comparison’ run of the *Diplodocus* model had the skull scaled to natural adult size, whereas in the ‘structural comparison’ run it was scaled so that the ratio of skull surface area to total applied muscle force was equal to that of the *Camarasaurus* model.

	min. element stress (MPa)	mean element stress (MPa)	max. element stress (MPA)
*Camarasaurus*	9.19 × 10^−8^	0.78	20.9
*Diplodocus*—ecological comparison	1.03 × 10^−11^	0.79	28.1
*Diplodocus*—structural comparison	2.01 × 10^−11^	1.12	37.6

#### (ii) Structural comparison

Comparison of von Mises stress plots from *Diplodocus* and *Camarasaurus* scaled so that overall surface area/applied force remains constant between them (removing effects of differential size and muscle forces) results in the *Diplodocus* skull experiencing higher mean and maximum element stresses than that of *Camarasaurus* (figure 3*a,b,e,f* and table 3). The regions of higher stress in *Diplodocus* remain largely restricted to the palate.

### (c) Biomechanical phylomorphospace

**Figure 4.**
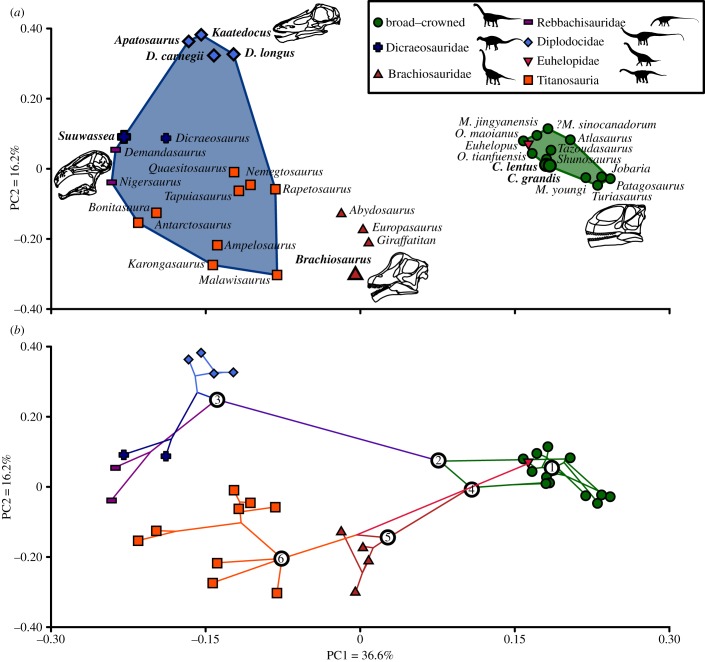
Craniodental biomechanical morphospace (‘functionspace’) plots for the Sauropoda. (*a*) Functionspace plot showing the distribution of the 35 included taxa on PC axes 1 and 2. Convex hulls used to illustrate the relative areas of biomechanical morphospace occupation of the ‘broad-crowned’ (in green) and ‘narrow-crowned’ (in blue) morphotypes. Taxa labelled: *C.*, *Camarasaurus*; *D.*, *Diplodocus*; *M.*, *Mamenchisaurus*; *O.*, *Omeisaurus*. Taxa from the Morrison Formation are indicated with enlarged points and labels in bold. Skulls of representative taxa found at extreme positive or negative PC values are illustrated—clockwise from top: *Kaatedocus* (adapted from [12]), *Turiasaurus* (adapted from [64]), *Brachiosaurus* (adapted from [65]) and *Nigersaurus* (adapted from [66]). (*b*) Phylomorphospace produced from projecting an informal supertree of the Sauropoda (see electronic supplementary material, §7). The position of *Tazoudasaurus*, the most basal included taxon, is marked as 1. Other numbers refer to the following nodes: 2, Neosauropoda; 3, Diplodocoidea; 4, Macronaria; 5, Titanosauriformes; 6, Titanosauria.

PC axes 1 and 2 together account for more than 50% of the total variance (figure 4). PC axis 3 accounts for a further 7.4%, after which variance scores reduce to less than 1% in PC axes 10 and above (for characters, character loadings and other PC axes, see electronic supplementary material, §§2–6). PC1 is primarily associated with characters correlated with maximum bite force such as toothrow length, posterior mechanical advantage of the jaw and adductor chamber size. More positive PC1 values relate to greater bite forces. PC2 is associated with deflection and expansion of the jaw joint, robustness of the mandible, and characters of the teeth. More negative PC2 values refer to more robust mandibles with occluding dentitions, whereas positive values reflect jaws with procumbent, non-occluding dentitions and an elongated articular glenoid, which would have permitted significant translational movements.

‘Broad-crowned’ taxa are restricted to positive values of PC1, whereas ‘narrow-crowned’ forms are restricted to negative PC1 values. Mapping of phylogeny (see electronic supplementary material, §7 for detail on the phylogeny used) shows that these groupings are non-monophyletic, with titanosaurs and diplodocoids showing convergent occupation of negative regions of PC1. However, these taxa are widely distributed in PC2 and still occupy significantly different regions of biomechanical morphospace (see electronic supplementary material, §5 for pairwise comparisons). Brachiosaurids occupy an intermediate position between ‘broad-crowned’ and ‘narrow-crowned’ forms.

## 4. Discussion

Biomechanical modelling demonstrates that *Camarasaurus* was capable of exerting much greater bite forces than *Diplodocus* through its more mechanically efficient skull, greater overall adductor muscle mass and a greater relative contribution of the external adductor muscle group. In addition, the skull of *Camarasaurus* is ‘stronger’ under static biting than that of *Diplodocus*, even after correcting for size. Nevertheless, peak and mean element stresses and overall stress distribution between the two remain comparable, and the skull of *Camarasaurus* may have also been more robust owing to spatial constraints resulting from its larger tooth roots [67]. The observed differences in bite force and cranial robustness indicate that *Camarasaurus* would have been able to crop harder foodstuffs than *Diplodocus*, which would have had a more restricted diet and/or engaged in less oral processing. This is consistent with tooth microwear evidence demonstrating a coarser diet in *Camarasaurus* than *Diplodocus* [34,68,69]. These results are also consistent with reconstructed feeding heights [34,67–71] (but see [72]) and higher relative tooth replacement rates in *Diplodocus* [35,67], which suggest that *Camarasaurus* may have been a more generalized browser on harder or potentially even woody material, whereas *Diplodocus* would have specialized on softer (but abrasive) foodstuffs such as horsetails and ferns [35,69,73]. Despite a weak bite force, the concentration of stresses within the palate of *Diplodocus*, which is relatively robust due to the expansion of the pterygoids, suggests that its unusual skull morphology is adapted towards the resistance of feeding-related loads (see also [36]). The larger-moment arms (due to ventral distension of the basal tubera) and greater relative importance of the ventroflexive craniocervical musculature in *Diplodocus* imply that ventrally directed movements of the head may have been especially important. These could have supplemented the weak bite forces, enabling severance of plant material gripped by the teeth, through rotation of the head [36] or ‘branch-stripping’ [34,36] (see electronic supplementary material, §11). During branch-stripping plant material would have been raked by the anterior tooth-comb as the head was pulled posteriorly [34,36] by ventroflexion of the neck. Contraction of the craniocervical musculature—particularly the ventroflexors—would have been important for maintaining head attitude during such movements. The short cervical ribs of diplodocoids relative to other sauropods would have permitted greater flexibility of the neck [34,74,75], as required for such feeding motions.

The observed functional separation between *Camarasaurus* and *Diplodocus* is reflected in craniodental biomechanical morphospace, in which they occupy opposite extremes of the total sauropod functional variance. Other Morrison Formation taxa are also widely distributed (figure 4*a*), demonstrating biomechanical differences that could have enabled niche partitioning between them. *Camarasaurus* shows the development of characters associated with high bite forces and a robust mandible. *Brachiosaurus* also demonstrates relatively high mechanical advantage of the jaw, and it occupies an intermediate position between *Camarasaurus* and ‘narrow-crowned’ taxa (figure 4*a*). Together with its ‘precision-shear’ bite, this indicates less oral processing potential in *Brachiosaurus* than *Camarasaurus*, and potentially the cropping of thinner branches. Diplodocoids are restricted to negative values of PC1, associated with low bite forces. Diplodocids are widely separated from other diplodocoids such as *Suuwassea* (figure 4*a*) by their procumbent dentitions, posteriorly inclined musculature and loss of dental occlusion. This study therefore provides the first quantitative support for previous assertions of niche partitioning on the basis of craniodental anatomy [32–34,69], and corroborates tooth microwear data [34,68,69].

The two broad anatomical craniodental morphotypes found in sauropods [35] are also found to be distinct within the biomechanical morphospace, although neither is monophyletic. *Euhelopus* converges with non-neosauropod sauropods and *Camarasaurus* in a relatively narrow ‘broad-crowned’ region of morphospace defined by characters associated with relatively high bite forces and interdigitating tooth occlusion. The ‘narrow-crowned’ diplodocoids, *Antarctosaurus*, *Bonitasaura* and nemegtosaurid titanosaurs all show functional convergence in characters correlated with relatively low bite forces and more gracile skulls. However, ‘narrow-crowned’ taxa also exhibit a much wider overall distribution within biomechanical morphospace than ‘broad-crowned’ forms and cannot be stereotyped as pertaining to a single uniform functional grade. This analysis is unusual in the inclusion of both cranial and mandibular biomechanical characters. Previous biomechanical disparity analyses [60,76–78] have included only mandibular characters due to the multiple roles of the skull, which could potentially influence feeding-related signals. However, data from the cranium and mandible here demonstrate a concordant signal, reinforcing the conclusions that could be drawn from either alone.

The niche partitioning between Morrison Formation taxa demonstrated herein provides a mechanism to support the high-diversity (but low-abundance [7]) sauropod communities in the resource-limited [7–10] and potentially low-quality vegetation-dominated (specifically low nitrogen [79]; but see [73,80]) Morrison Formation. Megaherbivore distribution is relatively independent of vegetation quality [1,2], with high megaherbivore diversity often coincident with poor forage [6]. Large size confers trophic advantages such as greater intake potential [81,82], increased fasting resistance [3,81], lower mass-specific metabolic rates [82–84] and increased ‘digestive priority’ towards fibre [83], even if not an increase in overall digestive efficiency [81–84]. Likewise, sauropod gigantism may represent an adaptation towards poor-quality forage [8,79,85]. However, plant productivity, and so ultimately rainfall, shows a strong positive correlation with megaherbivore diversity [1,2], so the sauropod dominance of the seasonally semi-arid Morrison [8–10] remains unique. Other advantages of gigantism in regions of patchily distributed or unreliable resources, such as increased locomotor efficiency [8,86] and increased fasting and drought resistance [8,81,86], may further explain the success of these extremely large herbivores in the Morrison environment. Nevertheless, as a general principle, dietary niche partitioning between sympatric taxa as demonstrated here is important in supporting high diversities of large herbivores regardless of taxon, even between large bulk-feeding herbivores with broad diets (see also [87]). It would therefore have been an integral mechanism in supporting high species richness in both dinosaur and mammalian megaherbivore communities of the Mesozoic and Cenozoic.

## Supplementary Material

Supplementary Information

## Supplementary Material

S1

## Data accessibility

A surface file of the *Camarasaurus lentus* skull and mandible reconstruction created for this study has been placed in the Dryad repository (doi:10.5061/dryad.8kd16).
