# Supplementary material for: Cranial biomechanics underpins high sauropod diversity in resource-poor environments
Source: Proc Biol Sci. 2014 Nov 22;281(1795):20142114. doi: 10.1098/rspb.2014.2114 (PMC4213629; doi:10.1098/rspb.2014.2114)
Supplement: Supplementary Information [file rspb20142114supp1.pdf]

# Supplementary information

## Cranial biomechanics underpins high sauropod diversity in resource-poor environments

Button, David J.[a,b], Rayfield, Emily. J.[a], and Barrett, Paul M.[b]

[a] University of Bristol School of Earth Sciences, Life Sciences Building, 24 Tyndall Avenue, Bristol, UK, BS8 1TP..

[b] Department of Earth Sciences, The Natural History Museum, Cromwell Road, London, UK, SW7 5DB.

**Table of contents**

1. **Taxon choice** ..... **2**
2. **Specimen measurements and taxon ages** ..... **2**
3. **Functional characters** ..... **3**
4. **Multivariate analysis** ..... **21**
5. **Multivariate analysis: additional results** ..... **24**
6. **Statistical tests of group separation in biomechanical functionspace** ..... **25**
7. **Phylomorphospace** ..... **26**
8. **Muscle reconstruction and force estimation** ..... **27**
9. **Finite-element model construction** ..... **31**
10. **Static biting analysis additional results** ..... **37**
11. **Branch-stripping analyses** ..... **39**
12. **Supplementary references** ..... **43**

## 1. Taxon choice

Measured specimens were those of all named sauropods (*sensu* [1, 2]) represented from craniodental remains sufficient to code them for at least 20% of the functional characters (see section 2). Taxa known only from teeth were rejected. Additionally data from the *nomen dubium* "*Astrodon*" were not included in disparity analyses as the association of relevant material is based only on assumed provenance [3].

Wherever possible data were only included from adult specimens. Ontogenetic status was assessed by the degree of sutural fusion of associated postcranial material, or where only crania are known, the degree of braincase suture fusion. In taxa where cranial material from multiple individuals is known (*Tazoudasaurus*, *Diplodocus*, *Camarasaurus* and *Europasaurus*) the overall size of the skull could also be used. However, due to the scarcity of sauropod cranial material measurements from subadult specimens were included for *Shunosaurus* and *Giraffatitan*.

## 2. Specimen measurements and taxon ages

A list of all specimens used, along with the raw measurements, is included in Supplementary Data, as are taxon occurrence ages and specimen references. All measurements were made in millimetres.

**Institutional Abbreviations used in Supplementary Data:**

**AMNH** – American Museum of Natural History, New York, USA; **ANS** - Academy of Natural Sciences, Philadelphia, USA; **CMNH** - Carnegie Museum of Natural History, Pittsburgh, USA; **CPSGM** - collections paléontologiques du Service géologique du Maroc, direction de la Géologie, ministère de l'Énergie et des Mines, Rabat, Morocco; **CPT** - Museo Fundación Conjunto Paleontológico de Teruel, Teruel, Spain; **CV** - Chongquing Museum of Natural History, Sichuan, China; **DFMMh** - Dinosaurier-Freilichtmuseum, Münchehagen,

Germany; **DINO** – Dinosaur National Monument, Jensen, Utah, USA; **FMNH PR** – Field Museum of Natural History, Chicago, USA; **IVPP** - Institute of Vertebrate Palaeontology and Palaeoanthropology, Beijing, China; **MACN** - Museo Argentino de Ciencias Naturales, Buenos Aires, Argentina; **MAL** – Malawi Department of Antiquities Collection, Lilongwe and Nguludi, Malawi; **MB.R.** - Humboldt Museum Für Naturkunde, Berlin, Germany; **MDE** – Espéraza Dinosaur Museum, Aude, France; **MDS** - Museo de Dinosaurios de Salas de los Infates, Sala de los Infantes, Burgos, Spain; **MMN** - Musée National du Niger; **MPCA** - Museo Provincial Carlos Ameghino, Río Negro, Argentina **MPEF** - Museo Paleontológico Egidio Feruglio, Trelew, Argentina; **MTM** – Musée de sciences de la Terre de Rabat, Morocco, **MZSP-PV** - Museu de Zoologia da Universidade de São Paulo, São Paulo, Brazil; **PIN** Russia Academy of Sciences, Moscow, Russia; **PMU** – Paleontological Museum, Uppsala, Sweden; **SMA** - Sauriermuseum, Aathal, Switzerland; **UA** – Université d' Antananarivo, Antananarivo, Madagascar; **UNSM** – United States National Museum, Washington DC, USA; **YPM** – Yale Peabody Museum, New Haven, USA; **ZDM** - Zigong Dinosaur Museum, Sichuan, China; **ZNM** - Zhejiang Museum of Natural History **Z.PAL** – Palaeobiological Institute of the Polish Academy of Sciences, Warsaw, Poland.

## 3. Functional characters

A total of 20 functional characters were measured from the mandible and skull of 35 sauropod taxa, to quantify the cranial functional disparity of the clade. Most analyses of functional disparity in cranial elements have focused on the mandible [e.g. 5-8] due both to its near-exclusive role in feeding (in contrast to the skull, which has multiple roles potentially resulting in functional compromise) and also to increase taxon coverage [6, 7]. However, as we are interested in the disparity of the entire cranium with respect to feeding-related specializations, characters of both the skull and mandible were included. This also allowed the inclusion of taxa known from good skull material but fragmentary or absent mandibular

remains. Characters were measured from published photographs and reconstructions, and where possible from CT reconstructions and direct examination of relevant material. All jaws and skulls were orientated equally for each measurement, with the level of the base of the toothrow horizontal for measurements taken in lateral or medial aspect. In taxa such as *Nigersaurus* and *Diplodocus* with highly abbreviated toothrows these elements were orientated so that the dorsal edge of the dentary/ventral edge of the maxilla lay at the horizontal. Measurements were taken in the program ImageJ (Rasband 1997-2012, http://rsb.info.nih.gov/ij/)

Sixteen of the characters are continuous measurements, and four are binary. The binary characters concern dental features, such as style of occlusion, that are difficult to quantify but show great variance within Sauropoda and have been inferred as distinguishing different functional classes [9-12]. Whilst most previous work on quantifying disparity has tended to use continuous characters exclusively, a similar combination of continuous measures and binary dental characters was utilized by Anderson *et al*. [6] in their investigation of early gnathostomes. The continuous characters represent a range of metrics associated with biomechanical performance and/or feeding ecology. The measurements required for each character are illustrated below on schematic images of the skull and mandible of *Camarasaurus lentus* (reconstructed and redrawn from CMNH11338, both from the Avizo reconstruction presented within and from [13]), with other taxa where appropriate.

### *Continuous characters*

#### (C1) Skull length

In herbivorous taxa increased body size expands foraging abilities, due to both the positive relationship between size and bite force [e.g. Wroe *et al*., 2005] and the inverse relationship between minimal acceptable diet quality and body size [e.g. Clauss *et al*., 2013].

Additionally, in nonchewing herbivores such as sauropods, skull size directly affects gape size and bite volume and hence the maximum size of fodder that can be ingested and foraging behaviour. Sauropods utilized the entire toothrow for cropping, with no or minimal oral processing [Christiansen, 1999; Upchurch & Barrett, 2000; Hummel & Clauss, 2011; Sander *et al*., 2011], and so gape size, jaw area and bite volume would have been the primary constraints acting upon their food intake rate [Christiansen, 1999]. Sauropods appear to have lost cheeks during their phylogenetic history [Barrett & Upchurch, 20007; Upchurch *et al*., 2007] potentially to increase gape size in association with bulk-feeding and increase intake rates [Barrett & Upchurch, 20007; Upchurch *et al*., 2007; Sander *et al*., 2011].

Skull length was measured along the ventral margin of the skull from the anterior tip of the skull to the anterior edge of the quadrate (figure S1). This measurement was chosen over the total skull length as it can be estimated from the mandible in taxa from which the cranium is not known. In such cases it was taken as the distance from the anterior tip of the dentary to the anterior margin of the articular glenoid. Additionally, this measure serves as a proxy for gape size, which becomes relatively decoupled from total skull length in diplodocoid taxa due to dorsoposterior rotation of the occiput, translational movement at the jaw joint and the shortening of the mandible.

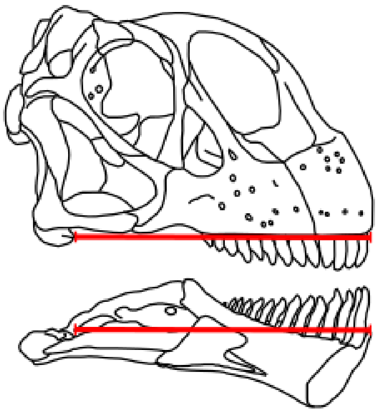

**Figure S1:** Measurements taken for the length of the skull ventral margin. Top: measurement of the character from the skull in lateral view, from the anterior tip of the snout to the anterior edge of the quadrate condyle. Bottom: alternative measurement of the character from the mandible in medial view, from the anterior tip of the dentary to the anterior margin of the articular glenoid. The latter measurement was used in taxa from which only mandibular remains are known.

**(C2) Anterior mechanical advantage of the mandible**

The mandible of vertebrates can be modelled as a third-order lever [14-16] where the mandibular musculature provides an input force, acting about the articular joint to exert an output force at the biting tooth. A simple measure of the efficiency of such a system is its Mechanical Advantage (MA, [15]) the ratio between the inlever and outlever, which represents the proportion of the input force that is transferred to the bite point. In a vertebrate jaw in lateral view the inlever can be approximated as the distance from the jaw adductor muscle attachment to the articular joint, and the outlever likewise approximated as the distance from the articular joint to the biting tooth. Although a simplification of a complex system involving multiple muscles of differing lines of action [16], MA is known to correlate with diet in extant fish [15-17] and the measure has been widely applied to extinct taxa including dinosaurs [18]. MA varies inversely with the speed of jaw closure and is often thought to increase in herbivorous lineages where speed of closure is no longer important (e.g. [19]). Stayton [19] found that although herbivorous lizards do not show overall convergence in jaw morphology, they do show convergence in increased mechanical advantage.

The MA at the anterior-most tooth position, furthest from the fulcrum, represents the lowest potential MA in the jaw. The inlever was measured from the centre of the jaw articulation to

the middle of the attachment area of the adductor musculature along the dorsal surface of the surangular, as in Anderson *et al*. [7] and Stubbs *et al*. [8]. This site was chosen due to the importance of the relatively efficient external adductors in static biting, and also that as many of the measured mandibles were known only in lateral view medial muscle attachment sites are more ambiguous. For simplicity, the resultant line of action of the muscle force was treated as lying perpendicular to the inlever.

The outlever was measured from the jaw articulation to the dentary at the point of the biting tooth. This position was chosen instead of at the tip of the biting tooth itself to allow comparison between specimens where some are known only with broken, missing or immature teeth, as in [15, 16]. Technically, outlever length will vary throughout biting, shortening during jaw closure and lengthening during jaw opening. However, this relative effect will influence all jaws, so only a single measurement on the jaw in horizontal orientation (closed- and so maximum outlever length) was made to avoid redundancy.

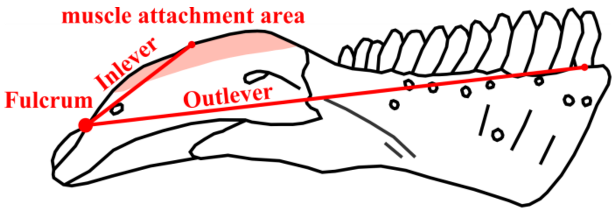


**Figure S2:** Measurements taken for the calculation of C2, anterior mandibular mechanical advantage.

### (C3) Posterior mechanical advantage of the mandible

The MA at the posteriormost bite point represents the greatest possible MA along the toothrow. It was calculated in the same manner as above, but with the outlever as the distance from the jaw articulation to the dentary at the point of the posteriormost tooth.

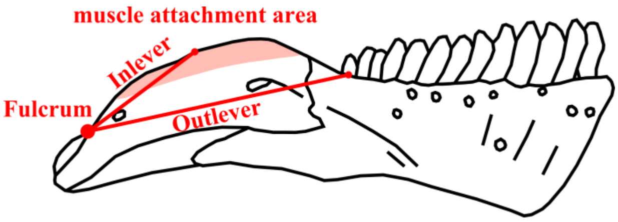


**Figure S3:** Measurements taken for the calculation of C3, posterior mandibular mechanical advantage.

### (C4) Articular offset of the jaw/jaw length

The position of the articular joint influences the occlusal pattern of the teeth. If the jaw joint lies in line with or close to the line of the toothrow the teeth will occlude in a scissor-like pattern, with the posterior teeth coming into occlusion first. In contrast, if the jaw joint is significantly offset from the level of the toothrow the teeth will meet in simultaneous occlusion. In extant mammals the former condition is seen in carnivores whereas the latter is typical of herbivores where simultaneous occlusion is important for the processing of plant matter [21]. Similarly, many extinct herbivorous groups demonstrate a jaw joint offset from the level of the toothrow [22] including many sauropods [11], so that an offset jaw joint is often taken as a general osteological correlate of herbivory [22].

The articular offset was measured by drawing a line level with the tooth-bearing portion of the jaw (the level of the toothrow itself was not used for the same reasons as referred to above) and then measuring the distance to the articular along a line drawn perpendicular to this (figure S4). This measurement was then divided by the overall jaw length to standardize the measurement for size. In taxa where the lower jaw is unknown this was estimated by the ventral offset of the quadrate articular condyle relative to the level of the maxillary ventral margin, divided by skull length.

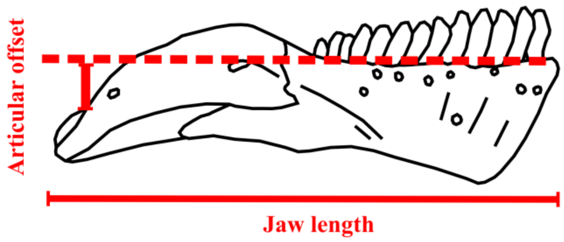


**Figure S4:** Illustration of the measurements taken to calculate C4, articular offset/jaw length.

### (C5) Relative length of the toothrow

A longer toothrow will result in a greater total variance in speed and power of a bite across the jaw. Additionally, it signifies a greater total area available for the cropping of vegetation. Toothrow length varies markedly within sauropods, with the convergent development of very short toothrows restricted to the anterior end of the jaw in multiple taxa. The relative length of the toothrow was calculated by taking the total length from the anterior-most to posteriormost tooth position of the mandible, and dividing it by the total mandible length (figure S5). In some sauropods the upper and lower toothrows are mismatched in length; the relative length of the lower toothrow was chosen as in such instances it is always the shorter, so any successive teeth in the upper toothrow will not be participating in occlusion.

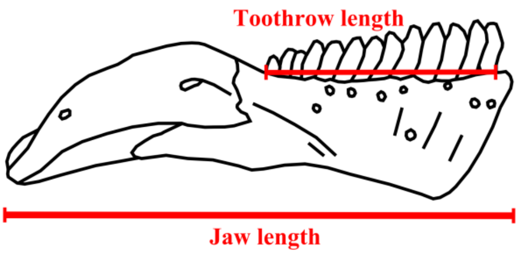


**Figure S5:** Illustration of the measurements taken for C5, the relative length of the toothrow.

### (C6) Maximum mandible height$^3$/mandible length

The mandible can be modelled as a beam, where its flexural stiffness will be proportional to the second moment of area ($I$), a measure of the distribution of material around the centroid

of the cross-sectional of a beam [23, 24]. It has been used as a proxy for resistance to bending of the mandible in multiple groups [e.g. 25, 26], including archosaurs [8, 27]. However, calculation of the second moment of area requires knowledge of the cross-section of the jaw, and as many of the specimens used in this study have only been figured in lateral view this was not possible.

In calculation of $I$ it is the cross section dimension along the axis of the load that is most important [7, 24]. In a jaw the primary load will be in the dorsoventral plane, so the height of the jaw can potentially serve as a functionally relevant proxy for resistance to bending under these loads [7], and height$^3$ will vary in proportion with $I$. Hence, the maximum dorsoventral height of the mandible was measured, cubed, and divided by the total length (figure S6). As a result, this measure is not dimensionless. However, size is important in mechanical performance, and structurally 'inefficient' structures can compensate simply by being larger [e.g. 28]. Unusually, sauropod mandibles tend to become deeper anteriorly, so the deepest region lies close to the symphysis, inferred as associated with a strengthening of the tooth-bearing portion of the jaw against loads associated with cropping behaviours [11].

Using the height of the jaw as a proxy for flexural stiffness in this manner does assume both a consistent width and uniform material across all jaws. Although both of these are ultimately inaccurate, if they are broadly consistent across the taxa being investigated jaw height can still serve as a reasonable comparative metric between taxa (see Anderson *et al.*, [7], who utilized this character in their analysis of basal tetrapods). This is generally the case amongst sauropods, although is potentially problematic for the aberrant taxon *Nigersaurus*, in which although the mandible is comparatively deep in lateral view parts of the mandibular elements themselves are around 1mm thickness.

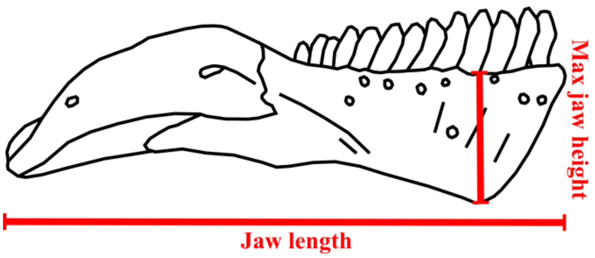


**Figure S6:** Illustration of the measurements taken for C6, the maximum height of the mandible/mandible length.

### (C7) Average mandible height$^3$/mandible length

As with Anderson *et al*. [7]'s analysis of the disparity of basal tetrapods the average height of the mandible/mandible length was also taken as a proxy for dorsoventral flexural stiffness. The average height of the mandible was calculated by measuring its area (minus the dentition) in lateral view, and dividing it by the length of the mandible. This value was cubed and then divided by the length of the mandible again, in a similar manner to the above character.

### (C8) Maximum symphyseal length/mandible length

Although the morphology of the mandibular symphysis is highly disparate within Archosauria, all sauropods retained the plesiomorphic symphyseal condition of simple abutting, unfused plates [29]. Nevertheless, in archosaurs the symphysis is important in withstanding a range of shear, bending and torsional stresses [30, 31] and transferring force between the working and balancing sides during asymmetric biting [30]. The dorsoventrally expanded symphysis of sauropods is inferred to represent an adaptation towards stresses related to cropping [11]. Walmsley *et al*. [31] demonstrated that linear measurements can accurately predict the behaviour of the symphysis under various loading conditions. Here the maximum length of the symphysis relative to the overall mandible length was taken as a proxy for its mechanical performance, after Anderson *et al*. [7]. The longest dimension of the

symphysis across the surface of the union of the jaws was measured and divided by the total jaw length (figure S7). It is worth noting that the orientation of this measured axis will vary between taxa. Other measures of symphysis size, primarily the symphysis length along the long axis of the jaw, were not included as the paucity of specimens figured in medial or dorsal view resulted in large amounts of missing data.

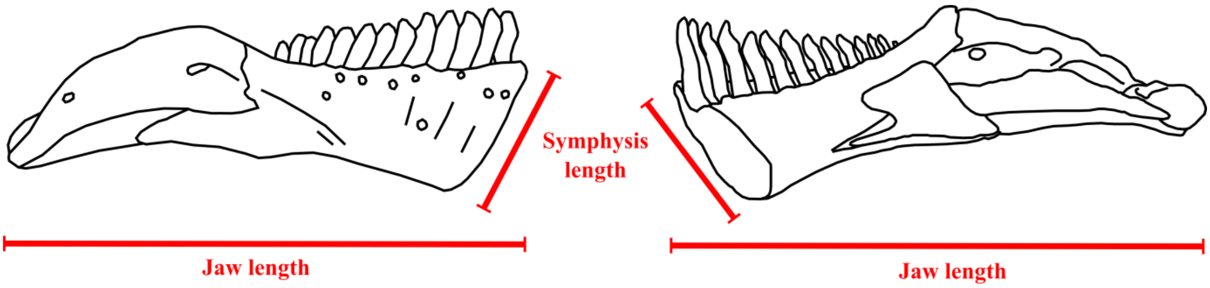


**Figure S7:** Illustration of the measurements taken for calculation of C8, mandibular symphysis maximum length/mandible length, in both lateral (left) and medial (right) views.

**(C9) Adductor fossa length/jaw length**

The total length of the muscle insertion area on the mandible (adductor fossa length) was divided by the total mandible length to give a proxy for the relative total area of muscle attachment (figure S8). This serves as a proxy for the size and, as muscle output force is proportional to cross sectional area, strength of the jaw musculature.

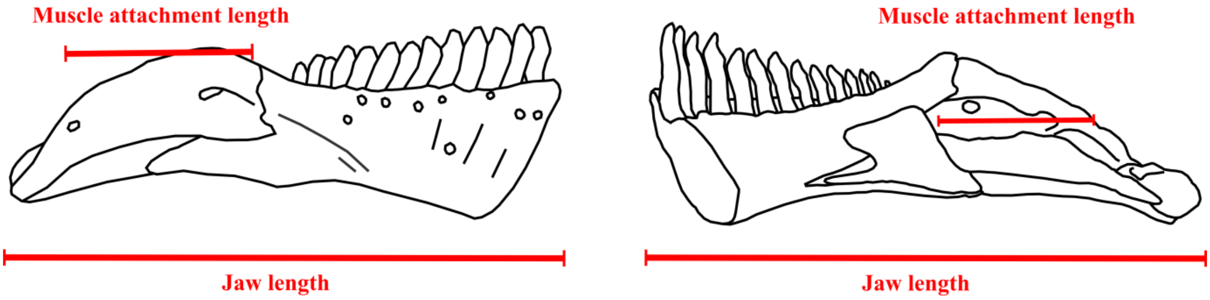


**Figure S8:** Illustration of the measurements taken for C9, the adductor fossa length/mandible length.

**(C10) Supratemporal fenestra anteroposterior principal axis length/skull length**

The temporal (m. adductor mandibulae externus and m. pseudotemporalis superficialis) muscles originate from the surfaces of the bones bordering the supratemporal fenestra in sauropsids [32]. Ideally, the size of the adductors would be constrained by measurement of the subtemporal fenestra, but the paucity of sauropod skulls figured in ventral view results in too small a taxon sample. Instead, the principal axes of the supratemporal fenestra were measured as they can be readily assessed in the more commonly provided dorsal and lateral views. Principal axes of the supratemporal fenestra were measured as a proxy for area. These separate measures were chosen over a single measurement of area as it allowed a measure of supratemporal fenestra size to be taken even in fragmentary specimens including only the skull roof.

The maximum length of the anteroposterior axis of the supratemporal fenestra, divided by the overall anteroposterior length of the skull (figure S9), was taken as a proxy of adductor muscle strength. This character was measured in dorsal view where possible, otherwise in lateral view. Comparison of measures of taxa from which both orientations are recorded indicated the measurements are equivalent.

**(C11) Supratemporal fenestra mediolateral axis/skull width**

Similarly, the maximum length of the supratemporal fenestra axis divided by the breadth of the skull (measured across the midpoint of the postorbital bar) was also taken as a proxy for adductor muscle strength (figure S9). This character was measured in dorsal view. Both C11 and C12 were necessary as some taxa (e.g. *Giraffatitan*) exhibit anterposteriorly short but very wide supratemporal fenestrae. Additionally as C11 can be calculated from a lateral view and C12 from an incomplete posterior skull, measurement of these separate characters permitted greater completeness relative to a single measurement of dorsal supratemporal

fenestra area. In some taxa lacking preserved postorbitals (*Suuwaasea*, *Ampelosaurus*, *Bonitasaura*) this character was estimated according to reconstruction of the postorbital and overall width based upon the width of the frontals and position of the posterolateral frontal-postorbital articulation.

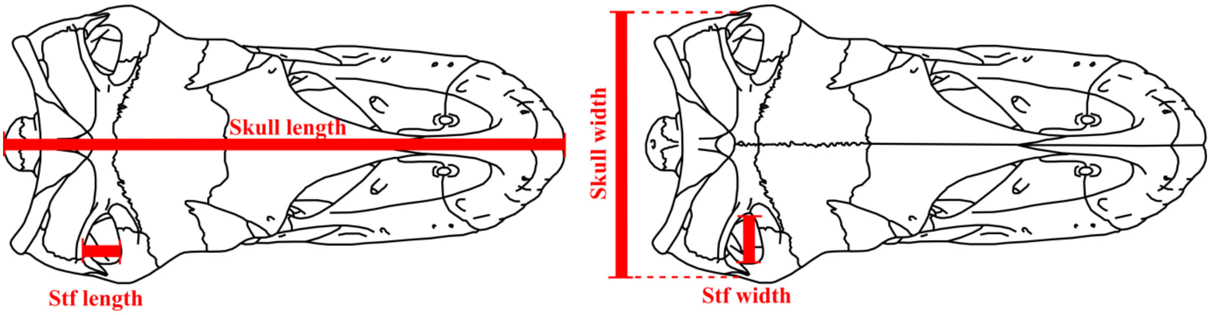


**Figure S9:** Illustration of the measurements taken for characters C10 (left) and C11 (right).

**(C12) Adductor muscle angle**

The line of action of the temporal adductor musculature lies along an angle to the vertical (theoretically varying between 0–90°). Consequentially, the resultant vertical bite force imparted will vary with the cosine to this angle. It hence follows that, for a given muscle force, the closer this angle is to vertical the greater the resultant vertical bite force when the jaws are near closed. The line of action of the temporal muscles varies markedly between sauropod taxa, with some taxa such as *Camarasaurus* demonstrating a near-vertical adductor chamber (with the muscles acting upon a more favourable line of action) whereas others such as *Diplodocus* demonstrate a strongly inclined adductor chamber at >45˚ to the vertical, which may be associated with propalinal jaw movement. To quantify this variance, the line of action of the temporal muscles was reconstructed along the middle of the area of insertion of the jaw to the middle of the postorbital bar on the articulated skull and jaws in lateral view. The angle from the vertical of this line of action was then measured (figure S10).

The aberrant taxon *Nigersaurus* is problematic in regards to this character as it has closed the supratemporal fenestrae and a bend in the quadrate blocks the line from the insertion area on the surangular to the temporal region [33]. Here we follow Sereno *et al*. [33] in assuming that this muscle mass must have shifted onto the quadrate, and measured the line of action accordingly.

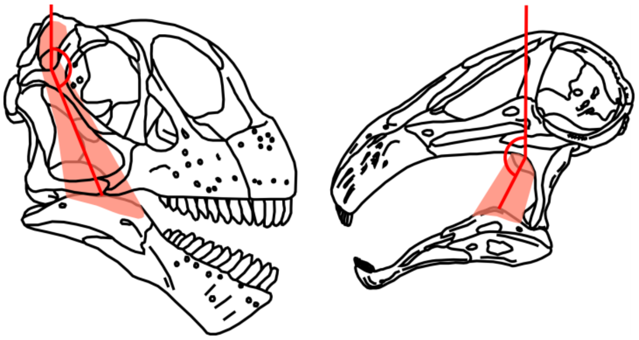

**Figure S10:** Illustration of the measurement of the angle of the temporal musculature from the vertical, for C12, in *Camarasaurus* (left) and *Nigersaurus* (right). For *Nigersaurus* the temporal muscle position, shifted onto the quadrate, of Sereno *et al*. [33] was used. *Nigersaurus* skull reconstruction modified from [33].

**(C13) Quadrate condyle length/articular glenoid length**

In many herbivorous taxa the anteroposterior length of the articular glenoid is elongated relative to the anteroposterior length of the quadrate condyle [22], allowing fore-and-aft movements of the mandible (propaliny) to be used in foraging or processing behaviours. The same is true of many sauropods, particularly the diplodocids, where it is thought to have been associated with specialized cropping behaviours [11, 12]. To quantify this the anteroposterior length of the quadrate condyle was measured and divided by the length of the articular glenoid. This provides the proportion of the glenoid filled by the quadrate, and so a measure of how much for-and-aft movement would have been possible (figure S11). Although ideally measured from a ventral view of the skull and dorsal view of the mandible (figure S11) this

character could also be measured from a lateral/medial view of the skull and medial view of the jaw, and estimated from a lateral view of the jaw where necessary.

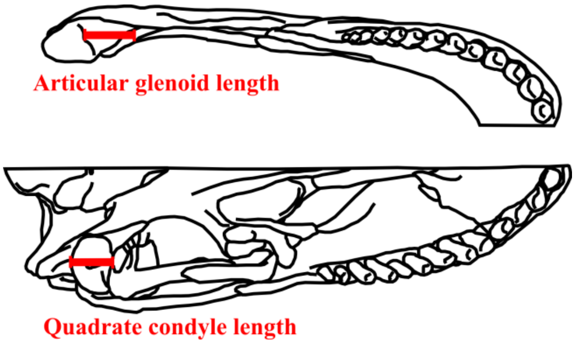


**Figure S11:** Measurement of the anteriorposterior length of the articular glenoid on the mandible in dorsal view (top) and quadrate condyle from the skull in ventral view (bottom) for C13.

**(C14) Premaxillary divergence angle**

The shape of the snout is correlated with feeding ecology in extant herbivores, with the general observation that nonselective grazers feeding on low, sward-like vegetation tend to have broader snouts, as opposed to the narrower snouts of more selective browsers [34-38]. Although a strict association of diet and snout shape is an oversimplification and a combination of proxies is more appropriate [39], numerous studies have found a similar relationship between snout shape and diet in extinct mammals [40-42], and such has often been inferred for herbivorous dinosaurs [e.g. 43]. Whitlock [44] utilized multiple measures of snout shape in his analysis of sauropod (primarily diplodocid) feeding and found a distinction in snout breadth between purported ‘grazers’ (*sensu lato*) and browsers, corroborated by dietary evidence from tooth microwear. One of the measurements employed by Whitlock [44] was the Premaxillary Divergence Angle (PMDA) the angle between a line drawn from the external edge of the midline premaxilla-premaxilla suture to the lateral edge of the premaxilla-maxilla suture and a horizontal line drawn from the midpoint of the snout, in

dorsal view (figure S12). This metric was chosen as it is relatively robust to incomplete or warped material, requiring only a complete premaxilla in dorsal view rather than a fully reconstructed anterior skull [44] and can be calculated from the width of the premaxilla in anterior view and length in lateral view using simple trigonometry. In the absence of preserved premaxillae in the taxa *Tazoudasaurus, Patagosaurus, Demandasaurus, Antarctosaurus* and *Bonitasaura* the PMDA can be estimated from the anterior attitude of the dentary in dorsal view, with the external jaw margin adjacent to the fourth dentary tooth (which would occlude with the final premaxillary tooth) marking the approximate position of the ventrolateral margin of the premaxilla.

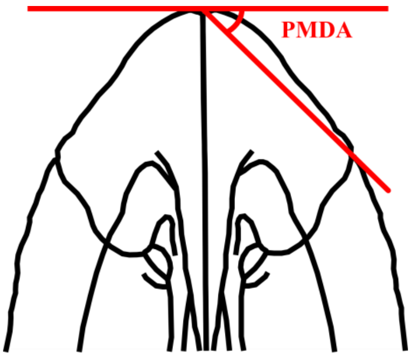


**Figure S12:** Dorsal view of the snout (premaxillae and anterior part of the maxillae), demonstrating the measurement of C14, the Premaxillary Divergence Angle (PMDA).

### (C15) Tooth angle

The angle of the long axis of the teeth relative to the jaw varies markedly in sauropods, with taxa such as *Shunosaurus* and *Nemegtosaurus* showing teeth approximately orthogonal to the jaw margin, with other taxa showing slight/moderate procumbency of the teeth and with *Diplodocus* showing a highly procumbent dentition that no longer can be brought into occlusion. Procumbent dentitions, especially highly procumbent ones, will be less effective at static biting as the inclination of the long axis with respect to the biting direction will result in

bending within the teeth; dentitions of varying procumbency probably represent specializations towards various raking and branch stripping behaviours [11, 45, 46].

The angle between the long axis and the tooth and a line at the level of the base of the toothrow was measured for all teeth present in life position in both the upper and lower toothrows of each specimen (figure S13). The mean of these values was then taken as the tooth angle. In some specimens where teeth are absent but damage allows the orientation of tooth roots and replacement teeth to be seen (e.g. *Apatosaurus*, CMNH11162) the angle of these were used to estimate the tooth angle.

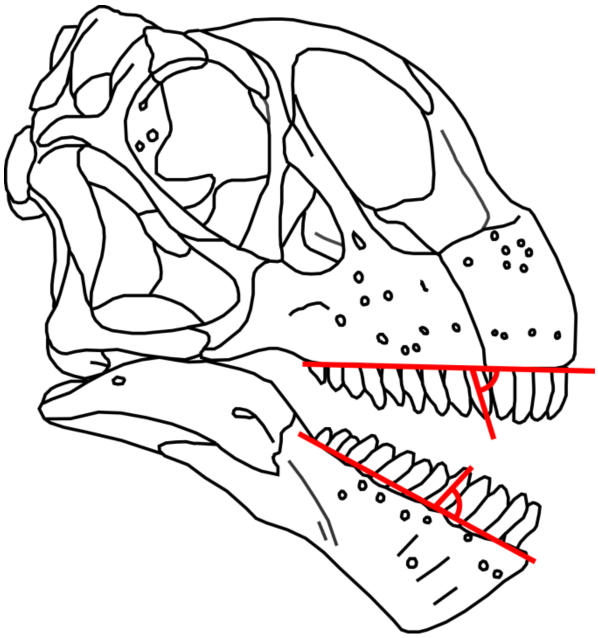

**Figure S13:** Illustration of the measurements conducted to calculate C15, the tooth angle. The angle from the level of the jaw for all present teeth, upper and lower, was measured with the mean then taken as the average tooth angle.

**(C16) Tooth slenderness index**

The slenderness index of sauropod teeth is the ratio of the height of the crown to the maximum breadth of the crown. Initially developed as a phylogenetic character [47] it has

since been used to classify sauropods into the ‘broad’ and ‘narrow’ crowned functional groups and trace the comparative diversity of each through time [12, 48]. ‘Broad-crowned’ teeth are more robust, and tend to show the development of heavy mesiodistal wear facets resulting from interdigitating occlusion. Narrow-crowned teeth in contrast are more gracile, and generally associated with either more precise shearing or an absence of occlusion.

***Discrete characters***

**(C17) Occlusion: absent (0) or present (1)**

The development of occlusion is an important adaptation towards specialized cropping of coarse foliage observed in eusauropods [49] and *Tazoudasaurus* [50, 51]; contrasting with the puncture-crushing dentitions of more basal sauropodomorphs [49]. The presence or absence of occlusion is clear from the presence or absence of tooth-tooth wear facets. However, the paucity of cranial material from basal sauropod taxa renders it currently unclear as to where within the basal Sauropoda tooth-tooth occlusion first appeared [49].

Within Eusauropoda, diplodocids secondarily lose occlusion [12, 45]. Although the style of occlusion varies between sauropods (see below), the presence of occlusion is still an important functional similarity between those taxa that do exhibit occlusion in comparison to those that do not, hence the inclusion of this character as well as C19 and C20.

**(C18) Interdigitating occlusion**

The plesiomorphic condition for sauropods, present in most ‘broad-crowned’ forms, consists of imbricating broad-crowned teeth meeting in an interdigitating occlusion, with each tooth occluding between two in the opposite jaw. This leads to the development of wear facets on the mesial and distal margins of each tooth.

**(C19) ‘Precision-shear’ bite**

Titanosauriform sauropods, at least *Nigersaurus* amongst rebbachisaurids and probably the dicraeosaurids [11, 12 although see 10] exhibit high-angled apical tooth-tooth wear facets on the lingual surfaces of upper teeth and the labial surfaces of lower teeth, producing a ‘chisel-like’ tip in each. This would have resulted from the teeth meeting one from the opposite jaw in precise one-to-one correspondence, rather than in an interdigitating fashion, along a relatively high-angled occlusional plane [11, 12]. This guillotine-like ‘precision-shear’ bite would have been effective at severing through stems and other plant material [11], but would be less suited to oral processing than the more plesiomorphic interdigitating-bite condition. Interestingly, many taxa bearing these dentitions specialized for slicing, but not processing, also show restriction of the toothrow to the front of the snout (resulting on lower maximum bite forces).

Whilst some such taxa (e.g. *Giraffatitan*, *Nemegtosaurus*) possess rare mesiodistal wear facets [11] the predominance of apical wear facets suggests that interdigitation would have been rare/insignificant, potentially only resulting from dental aberrations or where a newly emergent tooth came into opposition against two mature, elongate teeth in the opposite jaw.

**(C20) Presence of a self-supporting tooth battery**

The rebbachisaur *Nigersaurus* possesses a self-supporting tooth-battery with a highly elevated number of tooth positions, elevated tooth replacement rates and wear facets that are continuous from one tooth to the next. This results in the eruption of a series of teeth that became worn in unison as a single continual blade [33, 52]. Rebbachisaurid cranial material is rare, but the lack of similar transverse expansion and the presence of distinct alveoli in the dentary of *Demandasaurus* indicates that such a battery was absent in this taxon. Given the position of *Demandasaurus* as a closely related nigersaurine [53] it is possible that a dental battery was restricted to *Nigersaurus*. Although the introduction of a binary character for a

single taxon could be potentially problematical, *Nigersaurus* is clearly highly distinct from all other sauropods in multiple aspects of functional craniodental anatomy; hence the inclusion of such a character was deemed justified.

## 4. Multivariate analysis

The continuous biomechanical characters were *z*-transformed (standardized so that the mean of each character was 0, with a standard deviation of 1). These scores were then subjected to a Principal Coordinate Analysis (PCO), performed in PAST [54] to produce a multivariate biomechanical morphospace (‘functionspace’). The Gower Similarity index was used in PAST to compute similarity as it can applied to mixed data (containing both continuous and categorical data). Table S1 presents summary statistics for the first 10 PC axes.

**Table S1**

| Axis | Eigenvalue | % variance |
|---|---|---|
| 1 | 1.442 | 36.578 |
| 2 | 0.56619 | 16.239 |
| 3 | 0.21846 | 7.4133 |
| 4 | 0.14774 | 3.5628 |
| 5 | 0.12438 | 3.2475 |
| 6 | 0.10671 | 2.6365 |
| 7 | 0.077536 | 1.7826 |
| 8 | 0.064483 | 1.7019 |
| 9 | 0.055172 | 1.3406 |
| 10 | 0.042625 | 1.0713 |

**Table S1-** Summary statistics for first 10 PC axes, computed in PAST.

The resulting biomechanical variation is strongly captured by PC axes 1 and 2, which together account for over 50% of the variance. To investigate the changes in functional characters within the resulting functionspace the strength of correlation of each character with PC axes 1 and 2 was tested using the Spearman's Rank Correlation Coefficient, computed in PAST (table S2).

**Table S2**

| | r values and *$r^2$ values | | *p*- values | |
|---|---|---|---|---|
| | **PC1** | **PC2** | **PC1** | **PC2** |
| **C1** | 0.39154 **0.1533* | -0.18323 **0.03357* | **0.022802** | 0.79967 |
| **C2** | 0.25978 **0.06748* | -0.31499 **0.09922* | 0.14148 | 0.15999 |
| **C3** | 0.60344 **0.3641* | -0.33946 **0.1152* | **5.4088E-05** | 0.28005 |
| **C4** | 0.10717 **0.01148* | -0.46946 **0.2204* | 0.29086 | **0.008644** |
| **C5** | 0.74016 **0.5478* | -0.27786 **0.07721* | **2.8054E-10** | 0.83238 |
| **C6** | 0.022773 **0.0005186* | -0.48583 **0.236* | 0.47403 | **0.014212** |
| **C7** | 0.17875 **0.03195* | -0.39377 **0.1551* | 0.055792 | 0.094751 |
| **C8** | 0.32564 **0.106* | 0.14366 **0.02064* | 0.18472 | **0.03804** |
| **C9** | 0.45013 **0.2026* | -0.14394 **0.02072* | **0.011131** | 0.54762 |
| **C10** | 0.56793 **0.3226* | -0.16168 **0.02614* | **8.4703E-06** | 0.54896 |
| **C11** | 0.47863 **0.2291* | -0.23667 **0.05601* | **0.00024917** | 0.064776 |
| **C12** | 0.58828 **0.3461* | -0.36801 **0.1354* | **4.1176E-05** | 0.79699 |
| **C13** | 0.43166 **0.1863* | -0.49822 **0.2482* | **9.3363E-05** | **0.038729** |
| **C14** | 0.81479 **0.6639* | -0.27128 **0.07359* | **1.1109E-09** | 0.68277 |
| **C15** | 0.21127 **0.04463* | -0.67739 **0.4589* | 0.30026 | **1.2183E-08** |
| **C16** | -0.85501 **0.731* | 0.17092 **0.02921* | **1.6693E-12** | 0.97061 |
| **C17** | 0.357 **0.1274* | -0.76193 **0.5805* | **0.016819** | **0.00060712** |
| **C18** | 0.93173 **0.8681* | 0.15914 **0.02533* | **1.1719E-10** | 0.079657 |
| **C19** | -0.62212 **0.387* | -0.70859 **0.5021* | **0.00050366** | **1.028E-08** |
| **C20** | -0.24481 **0.05993* | -0.039456 **0.001557* | 0.092604 | 0.69824 |

**Table S2-** Strength of association of biomechanical characters with PC axes 1 and 2, computed in PAST. Metrics with a p value of <0.05 are highlighted in bold.

## 5. Multivariate analysis: additional results

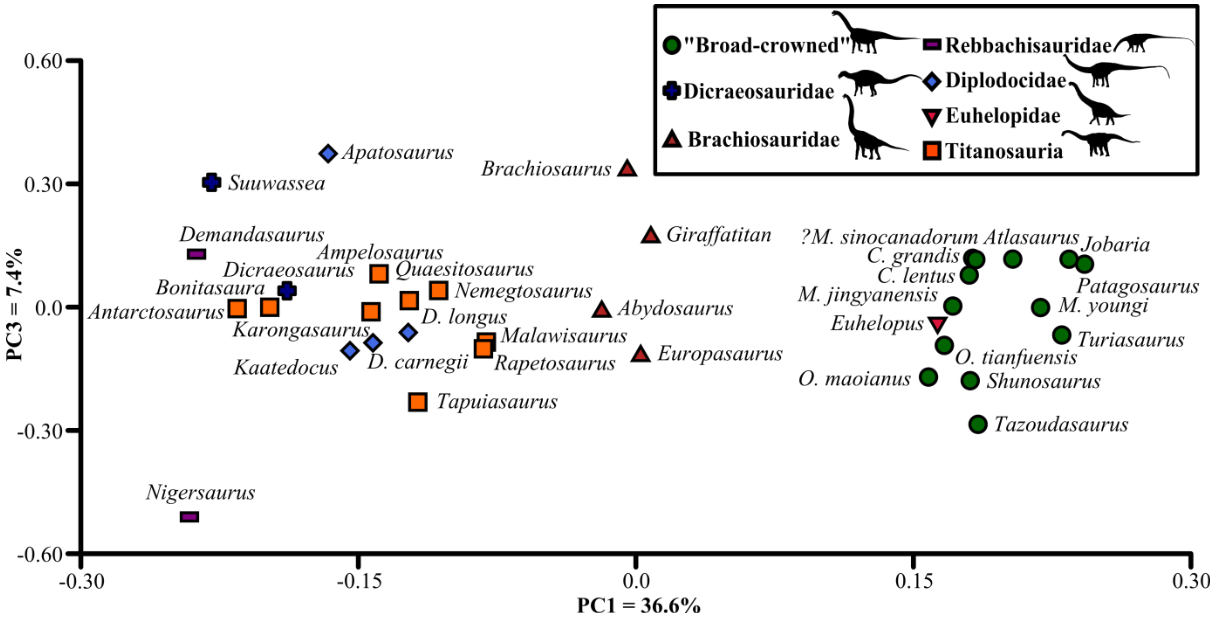


**Figure S14:** Biomechanical morphospace plot of PC axes 1 and 3.

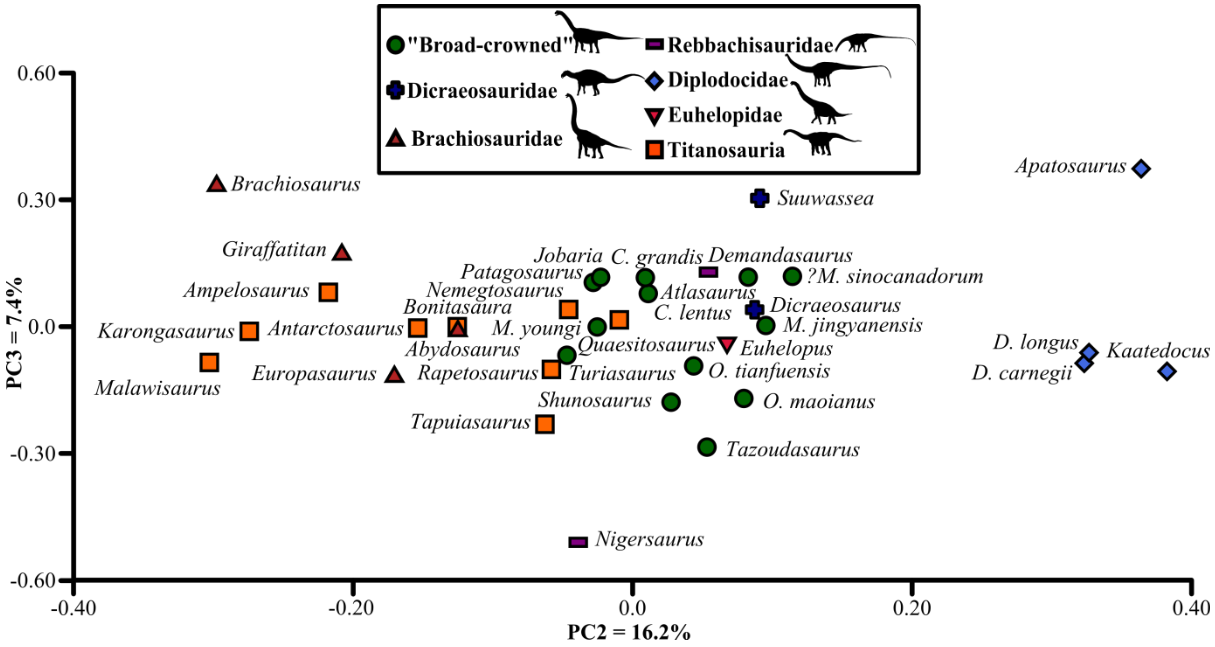


**Figure S15:** Biomechanical morphospace plot of PC axes 2 and 3.

## 6. Statistical tests of group separation in biomechanical functionspace

In order to test for the presence of functional convergence between ‘narrow-crowned’ diplodocoids and titanosaurs, and a functional distinction between a ‘broad-’ and ‘narrow-crowned’ forms the taxa were split into 1) basal ‘broad-crowned’ sauropods, 2) brachiosaurids, 3) diplodocoids and 4) titanosaurs (*Euhelopus* did not fit into any of these groups, and as a single point it could not be distinguished from any of the other groups. It is hence not reported on below). Differences in functionspace occupation of these groups was then tested with a non-parametric multivariate analysis of variance (npMANOVA) [55] with 100000 permutations conducted in PAST, utilizing PC scores of the first 18 axes (together accounting for 77.6% of the total variance). “Broad-crowned” and brachiosaurid taxa were found to be significantly distinct from all the other groupings (table S3). Additionally, diplodocoids and titanosaurs, despite showing the convergent occupation of similar areas of functionspace, were also found to be significantly different from each other and from all other groups (table S3).

**Table S3**

| | **"Broad-crowned"** | **Diplodocoidea** | **Brachiosauridae** | **Titanosauria** |
|---|---|---|---|---|
| **"Broad-crowned"** | | 0.0001 | 0.0003 | <1E-05 |
| **Diplodocoidea** | 0.0001 | | 0.0017 | 0.0097 |
| **Brachiosauridae** | 0.0003 | 0.0017 | | 0.0149 |
| **Titanosauria** | <1E-05 | 0.0097 | 0.0149 | |

**Table S3:** p-values of npMANOVA testing of functionspace occupation between sauropod groups.

## 7. Phylomorphospace

### Phylogeny

A time-calibrated informal supertree of the Sauropoda (defined as the least inclusive clade containing *Vulcanodon* and Eusauropoda [1, 2]) was constructed to project into the biomechanical morphospace (figure S16). Although the exact positions of several sauropod taxa (e.g. *Patagosaurus*, *Mamenchisaurus*, *Omeisaurus*, *Atlasaurus*) vary between competing matrices (e.g. [45, 58-61]), a largely resolved topology was produced with the relationships of basal sauropods based upon [51, 61, 62], those of diplodocoids upon [63, 64] and those of macronarians upon [62, 65-67]. These phylogenies were chosen on the basis of the taxa included and date of publication. The phylogenetically problematic Late Triassic putative sauropod *Lamplughsaura* [68] was not included here as both of the suggested phylogenetic positions for this animal [see 68] fall outside of the Sauropoda as defined herein.

Taxa were dated to the level of Standard European Stages, with the first and last occurrences taken as concordant with the start of the earliest stage and end of the latest from which they are known, respectively. These were used to produce a time-calibrated tree utilizing the timePaleoPhy function within the paleotree package [69] in R (R Core Team, 2013, R foundation for statistical computing, Vienna, Austria, http://www.r-project.org/). Trees were dated utilizing the 'minMax' argument, where an observed date is drawn randomly from a distribution bounded by the first and last occurrence dates. Branches were scaled as in the method of Brusatte *et al*. [70], where zero-length branches are avoided through equal "sharing" of time with a preceding non-zero length branch. A single dated informal supertree was then projected onto the first two PC axes of the biomechanical "functionspace" utilizing the phytools package [71] within R.

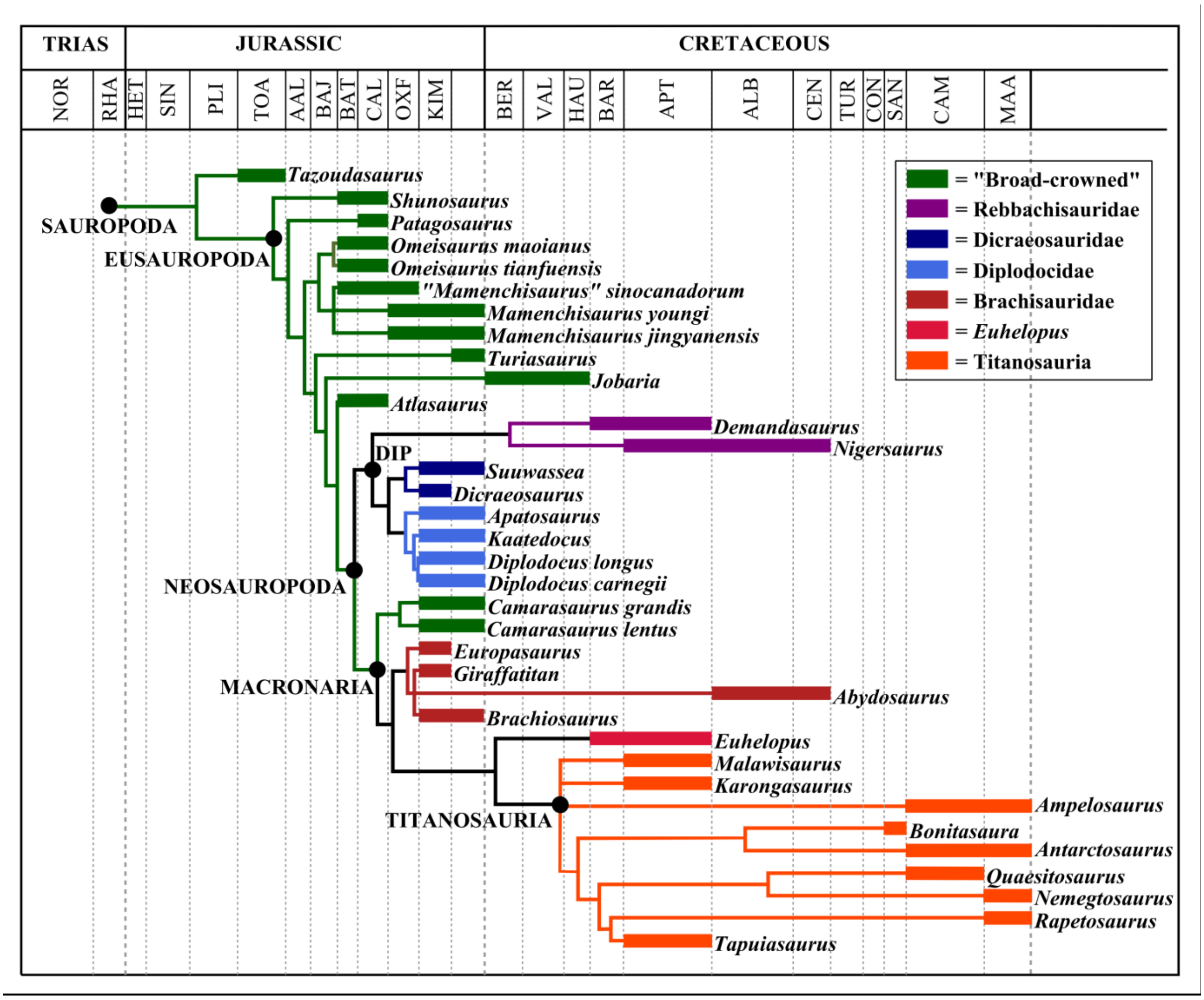


**Figure S16:** An informal supertree of the Sauropoda, demonstrating the relationships of the taxa included in this study (see text). The different groups plotted within the ‘functionspace’ (see figure 1) are color-coded as in figure 1; of these only the “broad-crowned” grade is not monophyletic. DIP = Diplodocoidea.

## 8. Muscle reconstruction and force estimation

### Muscle abbreviations

**Jaw adductors**- nomenclature follows Holliday [32].

m. AMEP- m. adductor mandibulae externus profundus; m. AMEM- m. adductor mandibulae externus medialis; m. AMES- m. adductor mandibulae externus superficialis; m. AMP- m.

adductor mandibulae profundus; m.PSTs- m. pseudotemporalis superficilias; m. PTd- m. pterygoideus dorsalis; m. PTv- m. pterygoideus ventralis.

**Craniocervical musculature**- nomenclature follows [72]. The occipital of the m. iliocastalis capitus was reconstructed after [72, 73]; it should be noted, however, that Tsuihiji [74] and Snively *et al*. [75] were dubious about this insertion in dinosaurs.

m. c.- m. complexus; m. i.c.- m. iliocastalis capitis; m. l.c.p.- m. longissimus capitis profundus; m. l.c.s.- m. longissimus capitis superficialis; m. r.c.v.- m. rectis capitis ventralis; m. s.c.- m. splenius capitis; m. t.c.- m. transversospinalis capitus

**Muscle force estimation**

Muscle forces were estimated according to the 'dry skull method' [76], where contractile force equals physiological cross-sectional area multiplied by the specific tension of the muscle. Although originally developed for use in mammals [76] the two main assumptions- that muscle cross-sectional surface area is proportional to contractile force and that muscle sizes can be accurately estimated from osteological remains alone- are equally applicable to dinosaurs [77]. Muscles were reconstructed in Avizo (Versions 6.3 and 7, FEI Visualization Science Group) on the basis of osteological correlates and topological relations [32] (figure S17). The total volume of each was then measured in Avizo using the material statistics module. This was then divided by the total length of the muscle as estimation of total fibre length to achieve the physiological cross sectional area (PCSA).

CMNH11338 represents a juvenile *C. lentus*. As *Camarasaurus* shows little ontogenetic change in the skull [4] an adult-sized skull model was obtained by linearly scaling-up the model of CMNH11338 by a factor of 1.8 in all directions so that it equalled the length of an adult *Camarasaurus* skull (DINO28, anteroposterior skull length =528mm [78]). Adult

muscle cross-sectional areas were then obtained by multiplying the cross-sectional areas calculated for CMNH11338 by 3.24 (the square of the linear increase in dimensions).

As the specific tension of the muscles of extinct taxa cannot be measured directly, an entire possible range was bracketed by using a range of specific tension measures (147 kPa - 392kPa) for vertebrate muscle [79]. Higher specific muscle tensions would generate increased muscle and bite forces, but the relative differences between the two models would remain the same. Table S4 compares the resulting range in muscle force values, and the resulting upper and lower bounds on bite force resulting from the finite-element models. A lower bracket on the jaw muscle volumes was provided by reconstructing the minimum possible volumes on the basis of the muscle insertions areas. These volumes, and the resulting bite forces, are compared in table S5. Even bite forces from the minimum possible *Camarasaurus* muscle volume reconstruction exceed those calculated from the maximum muscle volumes for *Diplodocus*. Craniocervical muscle volumes and forces are given in table S6.

**Table S4**

| | Muscle volume /$m^3$ | PCSA/$m^2$ (Muscle volume/muscle length) | Minimum muscle force/N (147kPa) | Maximum muscle force/N (392kPa) |
|---|---|---|---|---|
| ***Camarasaurus* (juvenile)** | | | | |
| m. AMES | 6.93E-05 | 4.66E-04 | 68.4 | 182.3 |
| m. AMEP | 3.51E-05 | 1.79E-04 | 26.3 | 70.2 |
| m. AMEM | 3.83E-05 | 2.46E-04 | 36.2 | 96.4 |
| m. PSTs | 2.51E-05 | 1.22E-04 | 17.93 | 47.8 |
| m. AMP | 4.33E-05 | 3.89E-04 | 57.13 | 152.5 |
| m. PTd | 4.73E-05 | 4.82E-04 | 70.85 | 188.9 |
| m. PTv | 6.51E-05 | 4.47E-04 | 65.71 | 175.2 |
| ***Camarasaurus* (adult size)** | | | | |
| m. AMES | 2.25E-04 | 1.51E-03 | 222.0 | 592 |
| m. AMEP | 1.14E-04 | 5.8E-04 | 85.26 | 227.4 |
| m. AMEM | 1.24E-04 | 7.97E-04 | 117.2 | 312.4 |
| m. PSTs | 8.13E-05 | 3.95E-04 | 58.07 | 154.8 |
| m. AMP | 1.40E-04 | 1.26E-03 | 185.2 | 493.9 |
| m. PTd | 1.53E-04 | 1.56E-03 | 229.3 | 611.5 |
| m. PTv | 2.11E-04 | 1.49E-03 | 219.0 | 584.1 |
| **Bite force/N** | | | **Anterior: 342.53** | **Anterior: 913.73** |

| | | | | |
|---|---|---|---|---|
| | | | **Posterior: 726.91** | **Posterior: 1938.96** |
| ***Diplodocus*** | | | | |
| m. AMES | 1.26E-04 | 4.47E-04 | 65.71 | 175.2 |
| m. AMEP | 3.66E-05 | 1.04E-04 | 15.29 | 40.77 |
| m. AMEM | 7.32E-05 | 2.44E-04 | 35.87 | 95.65 |
| m. PSTs | 8.29E-05 | 2.63E-04 | 38.66 | 103.1 |
| m. AMP | 5.8E-05 | 3.74E-04 | 55.08 | 146.6 |
| m. PTd | 1.18E-04 | 1.04E-03 | 152.9 | 407.7 |
| m. PTv | 1.77E-04 | 9.08E-04 | 133.5 | 355.9 |
| **Bite force/N** | | | **Anterior: 124.77 Posterior: 179.54** | **Anterior: 336.95 Posterior: 479.82** |

**Table S4:** Reconstructed jaw adductor muscle volumes and forces for *Camarasaurus* and *Diplodocus*.

## Table S5

| | Muscle volume /$m^3$ | Muscle force/N (392kPa) |
|---|---|---|
| ***Camarasaurus*** **(adult size)** | | |
| m. AMES | 2.07E-04 | 544.6 |
| m. AMEP | 1.07E-04 | 213.8 |
| m. AMEM | 1.20E-04 | 303.0 |
| m. PSTs | 7.48E-05 | 142.4 |
| m. AMP | 1.25E-04 | 439.6 |
| m. PTd | 1.32E-04 | 525.9 |
| m. PTv | 1.12E-04 | 309.6 |
| **Bite force/N** | | **Anterior: 794.73 Posterior: 1694.1** |
| ***Diplodocus*** | | |
| m. AMES | 5.45E-05 | 75.73 |
| m. AMEP | 3.10E-05 | 34.57 |
| m. AMEM | 3.51E-05 | 45.91 |
| m. PSTs | 2.61E-05 | 32.48 |
| m. AMP | 3.39E-05 | 85.61 |
| m. PTd | 3.69E-04 | 127.6 |
| m. PTv | 4.11E-04 | 82.57 |
| **Bite force/N** | | **Anterior: 235.5 Posterior: 335.4** |

**Table S5**: Minimum possible reconstructed jaw adductor muscle volumes and forces.

## Table S6

| | Occipital insertion area (/$m^2$) | Estimated muscle cross sectional area (/$m^2$) | Minimum muscle force/N (147kPa) | Maximum muscle force/N (392kPa) |
|---|---|---|---|---|
| ***Camarasaurus*** **(juvenile)** | | | | |
| m. c. | 1.54E-04 | 1.06E-04 | 15.58 | 41.55 |
| m. t.c. | 2.91E-04 | 3.17E-04 | 46.6 | 124.3 |

| | | | | |
|---|---|---|---|---|
| m. s.c. | 2.23E-04 | 3.28E-04 | 48.22 | 128.6 |
| m. l.c.s. | 1.81E-04 | 2.71E-04 | 39.84 | 106.23 |
| m. l.c.p. | 7.10E-05 | 1.22E-04 | 17.93 | 47.82 |
| m. i.c. | 2.59E-04 | 2.38E-04 | 34.91 | 93.3 |
| m. r.c.v. | 1.00E-04 | 1.13E-04 | 16.61 | 44.3 |
| ***Camarasaurus* (adult size)** | | | | |
| m. c. | 4.94E-04 | 3.43E-04 | 50.42 | 134.5 |
| m. t.c. | 9.43E-04 | 1.03E-03 | 151.4 | 403.76 |
| m. s.c. | 7.23E-04 | 1.06E-03 | 155.8 | 415.5 |
| m. l.c.s. | 5.86E-04 | 8.78E-04 | 129.1 | 344.2 |
| m. l.c.p. | 2.30E-04 | 3.95E-04 | 58.07 | 154.8 |
| m. i.c. | 8.39E-04 | 7.71E-04 | 113.3 | 302.2 |
| m. r.c.v. | 3.24E-04 | 3.66E-04 | 53.80 | 143.5 |
| ***Diplodocus*** | | | | |
| m. c. | 4.12E-04 | 5.11E-04 | 75.12 | 200.3 |
| m. t.c. | 5.30E-04 | 6.48E-04 | 95.26 | 254.0 |
| m. s.c. | 4.18E-04 | 5.56E-04 | 81.73 | 218.0 |
| m. l.c.s. | 4.00E-04 | 4.16E-04 | 61.15 | 163.1 |
| m. l.c.p. | 2.10E-04 | 2.42E-04 | 35.57 | 94.86 |
| m. i.c. | 1.17E-03 | 6.51E-04 | 95.70 | 255.2 |
| m. r.c.v. | 1.04E-04 | 2.66E-04 | 39.10 | 104.3 |

**Table S6:** Reconstructed craniocervical muscle volumes and forces for *Camarasaurus* and *Diplodocus*.

## 9. Finite-element model construction

The *Diplodocus* model utilized in this study is that of Young *et al*. [45], with the input muscle forces modified as explained above. The *Camarasaurus* model was created for this study, and is expanded upon below.

### *Camarasaurus* finite-element model convergence test results

To save on computing time the majority of *Camarasaurus* models run for the analyses within were of only 877796 elements. To ensure this was sufficient a number of elements to provide an accurate representation of the skull and its behaviour under loading, analyses were performed comparing the stress magnitudes and distributions of models with up to 2.4 times the number of elements (table S7). Maximum, minimum and average stresses remained similar between all these runs.

**Table S7**

| Number of elements | Minimum element stress /Mpa | Average element stress /Mpa | Maximum element stress /Mpa |
|---|---|---|---|
| 877796 | 9.19E-08 | 0.78 | 2.09E01 |
| 1463596 | 2.73E-08 | 0.80 | 2.3E01 |
| 2133313 | 0.00 | 0.79 | 2.14E01 |

**Table S7:** Summary of results for element number convergence tests performed for the *Camarasaurus* finite-element model. Increasing the element number, even by a significant amount, has minimal effect on average element stresses. Critically, in terms of the results, they remain similar to those of the 'ecological comparison' *Diplodocus* model and significantly less than those of the scaled 'structural comparison' *Diplodocus* model in all cases.

**Material properties**

Material properties were assigned to the tissues in the meshing software Hypermesh (Version 11, Altair). Finite-element modelling of extinct taxa is problematic as the true material properties of structures are unknown [80]. Additionally, although cranial bone is anisotropic [81] anisotropy cannot be reliably measured in fossil specimens. However, validation studies [82, 83] have demonstrated that patterns of stress and strain can be reliably predicted even in models utilizing approximated and isotropic material properties, even if absolute magnitudes cannot. This means that finite-element analysis can still serve as a comparative tool between different loading conditions and different models in extinct taxa, so long as the boundary conditions are maintained consistent between them.

In the absence of genuine material properties, those of histological analogues were used. To aid comparison the same properties were used here as by Young *et al*. [45] in their analysis of *Diplodocus*. Sauropods are typified by fast-growing Haversian bone [84]. As a result, the skull bone of both taxa was ascribed the material properties of bovine Haversian bone (Young's Modulus = 23.1GPa; Poisson's ratio = 0.29) [85]. This measure is based upon long bones (specifically, femora) and so is likely an over-estimate; as such the lowest value of the Poisson's ratio was used [45]. Dentine was ascribed a Young's modulus of 21GPa and Poisson's ratio of 0.31 [86] and enamel a Young's modulus of 80GPa and Poisson's ratio of 0.3 [87], again both as in Young *et al*. [45].

Unfortunately enamel and dentine could not be easily resolved in the CT scans of *Camarasaurus*, or modelled separately in the finite-element model. As a result, the teeth of *Camarasaurus* were modelled as a single tissue of composite material properties (Young's modulus = 50.5 GPa, Poisson's ratio = 0.305). Sensitivity analyses where the teeth were instead given the material properties of dentine and enamel [figure S17] demonstrate little deviance from those employing this composite value.

**Constraints**

Sensitivity analyses constraining six, eight and 10 teeth were performed for each taxon (figures S18, S19). Each constraining of successive teeth results in minor changes in the distribution and magnitude of stresses, but overall patterns of stress are similar to those observed in models with only four constrained teeth. The models were also fully constrained at the quadrates. The models here replicate a static bite; full constraint at the quadrates was required. It should be noted, though, that this is potentially unrealistic given the high capacity for propalinal movements in *Diplodocus*. All constraints were applied as a Diffuse Coupling Constraint (DCC)- a series of rigid links that spread the constraint over multiple nodes. This

reduces problems of localized very high forces correlated with point constraints- indeed, in both models peak stresses did not occur at a constraint. Use of a DCC spreads out any high forces associated with constraints more effectively than simply utilizing an equivalent number of individual constraints, where individual nodes that are proximal to multiple constraints may become over-constrained, resulting in very high localized stress peaks.

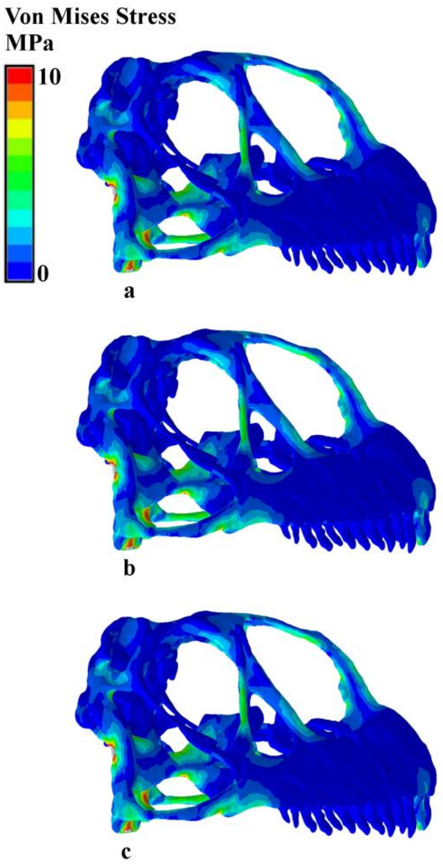


**Figure S17:** Comparison of analyses where the teeth were assigned intermediate properties (top), those of dentine (middle) and enamel (bottom). Overall stress patterns deviate little between these analyses, and only around the biting teeth.

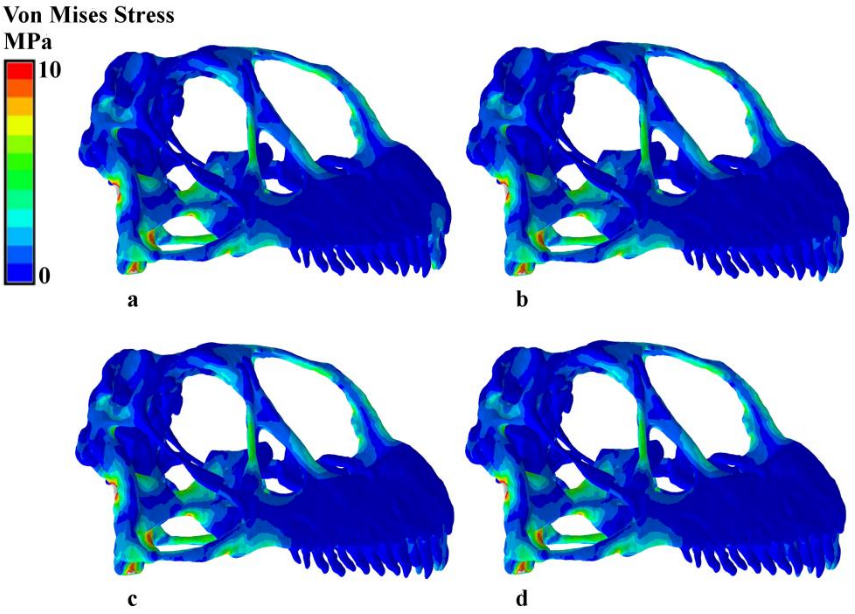


**Figure S18:** Comparison of sensitivity analyses of *Camarasaurus* constraining successively more of the anteriormost teeth. a) Four teeth constrained. b) Six teeth constrained. c) Eight teeth constrained. d) Ten teeth constrained. Although the magnitude and distribution of stress increases slightly with more constrained teeth, patterns of stress remain consistent.

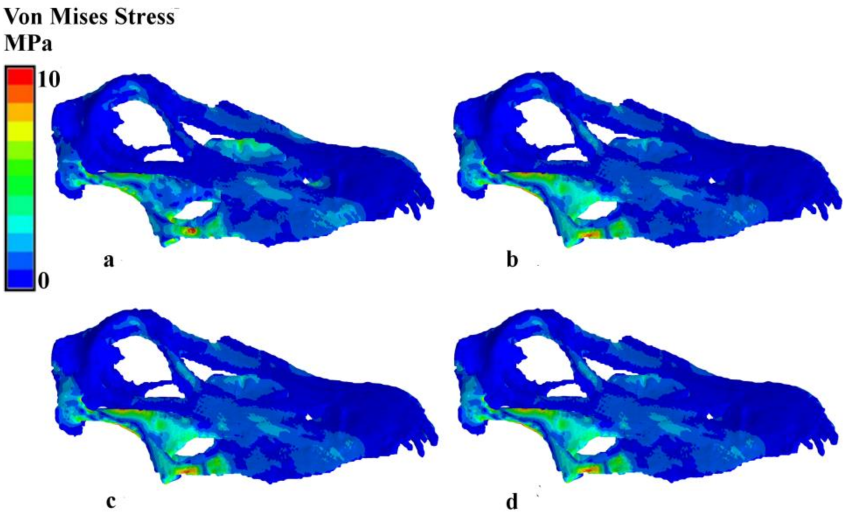


**Figure S19:** Sensitivity analysis results constraining successively more of the anteriormost teeth in *Diplodocus*. a) Four teeth constrained. b) Six teeth constrained. c) Eight teeth constrained. d) Ten teeth constrained.

For the static biting models the teeth were constrained against translation in the vertical (y) axis, the plane of biting, to simulate the teeth being brought into opposition against food/the opposing teeth in an orthal bite. This relatively relaxed constraint was chosen as analyses constraining the teeth in the x, y and z axes resulted in highly localized stresses in the biting teeth which were considered to be a result of over-constraint. Nevertheless, comparison of the results between these analyses differing in the degrees of freedom of the constraints applied to the teeth show very little difference (table S8). The *Camarasaurus* and both *Diplodocus* models all show higher peak stresses (in the biting teeth in all cases) but slightly reduced mean element stresses. Still, the relative performance of the three models, remains the similar- *Camarasaurus* and the "ecological comparison" *Diplodocus* model show very similar mean stresses, whereas the "structural comparison" *Diplodocus* model experiences notably higher peak and mean element stresses. The overall results are hence robust to the manner in which the constraints were treated.

| | Min element stress/MPa | | Mean element stress/MPa | | Max element stress/MPA | |
|---|---|---|---|---|---|---|
| Axes constrained at biting teeth | Y | X, Y, Z | Y | X, Y, Z | Y | X, Y, Z |
| *Camarasaurus* | 4.89E-08 | 9.19E-08 | 0.75 | 0.78 | 20.9 | 31.6 |
| *Diplodocus* - ecological comparison | 1.02E-11 | 1.03E-11 | 0.73 | 0.79 | 28.1 | 28.9 |
| *Diplodocus* – structural comparison | 1.37E-011 | 2.01E-011 | 0.99 | 1.12 | 37.6 | 39.1 |

**Table S8** – results from the static biting models comparing analyses constraining the biting teeth in the y-axis (the plane of biting) alone versus in the x, y and z axes. Overall results are very similar between the two, except for higher peak forces in the biting teeth in the analyses with constraint in all axes, considered here to be an artefact due to overconstraint.

## 10. Static biting analysis additional results

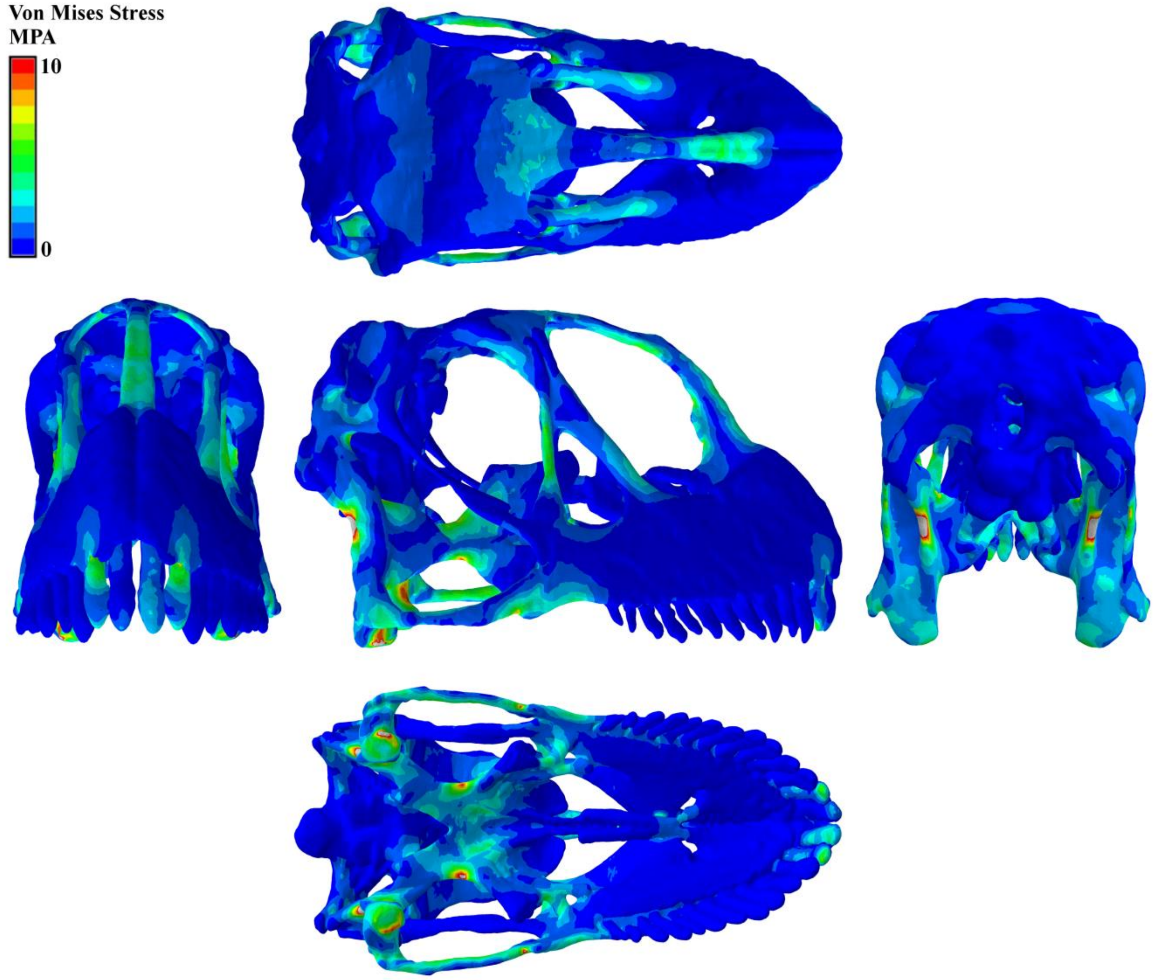


**Figure S20:** Additional views of the von Mises Stress contour plots FEA of the skull of *Camarasaurus lentus* (CMNH 11338), scaled to adult size.

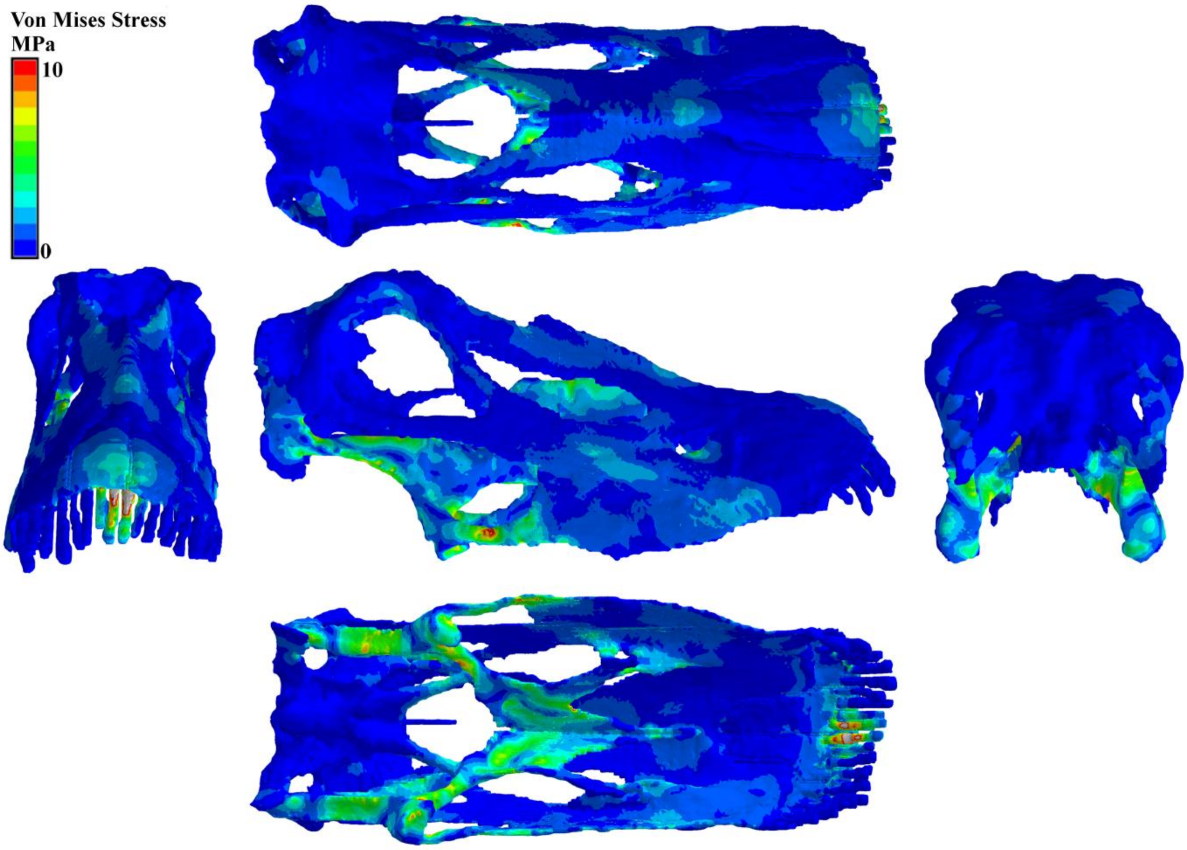


**Figure S21:** Additional views of the FEA results for the skull of *Diplodocus carnegii* (CMNH 11161).

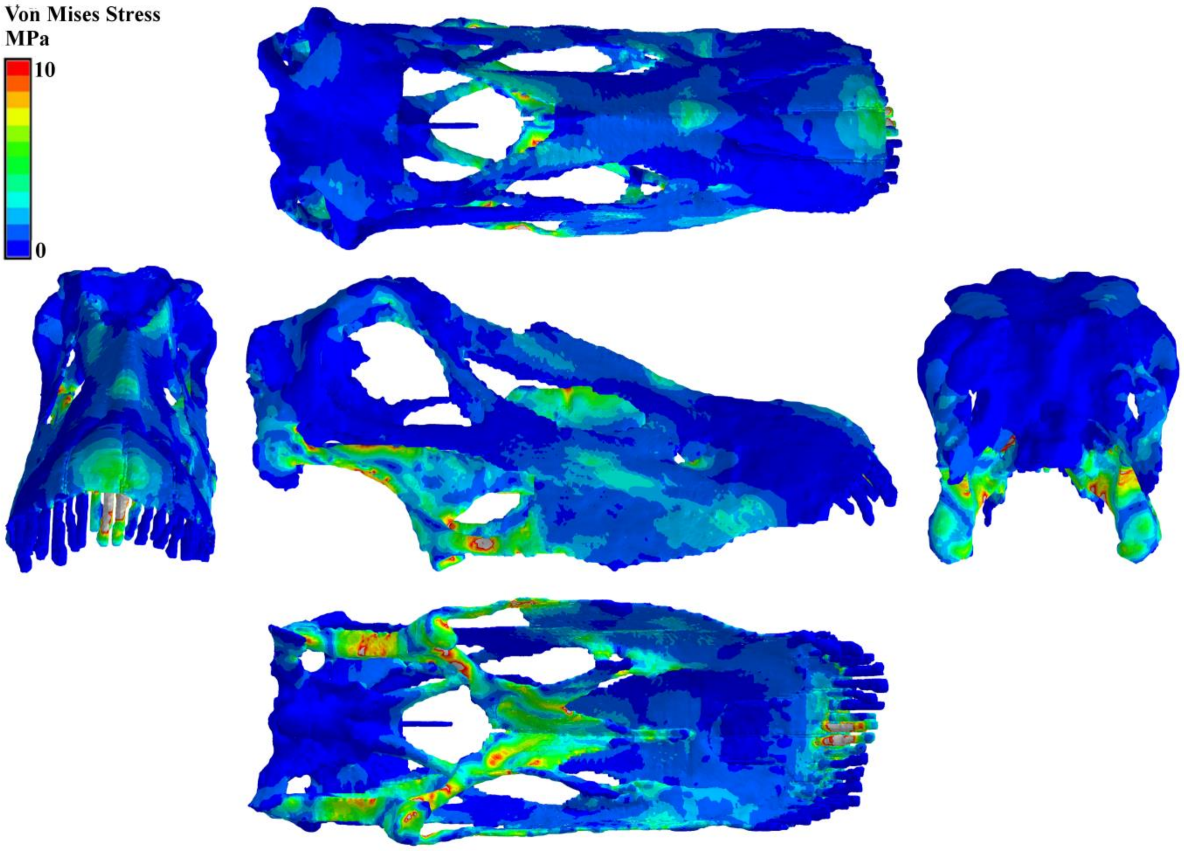


**Figure S22:** Additional views of the FEA results for the skull of *Diplodocus carnegii*, scaled so that overall applied force/skull surface area equals that of *Camarasaurus* (the "structural comparison").

## 11. Branch-stripping analyses

In addition to the static biting analyses, the 'branch-stripping' analyses of Young *et al*. [45] were also performed here, but with inclusion of the pull of the craniocervical musculature, wherein all muscle groups were modelled as contracting simultaneously. The skull of each taxon was modelled as if simultaneously biting and retracting the head in a posteriorly-directed motion, as if pulling to detach plant matter. Although there is no evidence of branch-stripping behaviour in *Camarasaurus*, tugging and wrenching motions would have been part of its foraging repertoire [11]. Additionally, given that *Camarasaurus* more closely

approximates the plesiomorphic sauropod condition, stripping behaviour was modelled here to provide a null model against which purported stripping-specific adaptations of *Diplodocus* could be tested.

The models were constrained at the anterior four biting teeth as above, and fully constrained at the occipital condyle. A stripping force was applied at the teeth equal to the shear strength of parenchyma (1E06 $Nm^{-2}$) multiplied by the area of the tooth in contact with the vegetation, after Young *et al*. [45]. Forces were calculated per tooth and then applied individually to each stripping tooth. Total stripping forces are given in table S9. The broad teeth of *Camarasaurus* result in very high stripping stresses; suggesting the absence of this behaviour in this taxon. Sensitivity analyses constraining and loading six, eight and 10 teeth were also performed figures S23, S24).

In *Diplodocus* the distribution and magnitude of stress resulting from branch-stripping is similar to that observed under static biting, so that it seems equally well-adapted to either [45] (figure S23). High stress is restricted to the condyle constraint point (where it is an artefact of the constraint) and in parts of the stripping teeth (figure S24). Elevated stress is also seen around the occipital condyle (especially at the ventral margin of the foramen magnum) and in the elongated basipterygoid processes.

In contrast, *Camarasaurus* performs significantly worse under branch-stripping than static biting (figure S24), as anticipated from the lack of specializations associated with such a behaviour that are seen in *Diplodocus* (the slender, procumbent dentition, the overlapping maxilla-dentary ‘pseudocheek’ etc. [see 11, 45, 46]) and the large forces applied to the teeth. Very high stresses are observed in the snout (figure S24), as a consequence of these very large forces acting upon the stripping teeth.

**Table S9**

| Taxon | Total tooth area/m$^2$ | Total applied branch stripping force |
|---|---|---|
| ***Diplodocus*** | | |
| 4 teeth | 1E-04 | 100 |
| 6 teeth | 1.5E-04 | 150 |
| 8 teeth | 2E-04 | 200 |
| 10 teeth | 3.5E-04 | 350 |
| ***Camarasaurus*** | | |
| 4 teeth | 3.71E-03 | 3712 |
| 6 teeth | 5.36E-03 | 5364 |
| 8 teeth | 7.02E-03 | 7016 |
| 10 teeth | 8.67E-03 | 8668 |

**Table S9: Branch** stripping forces calculated for each taxon.

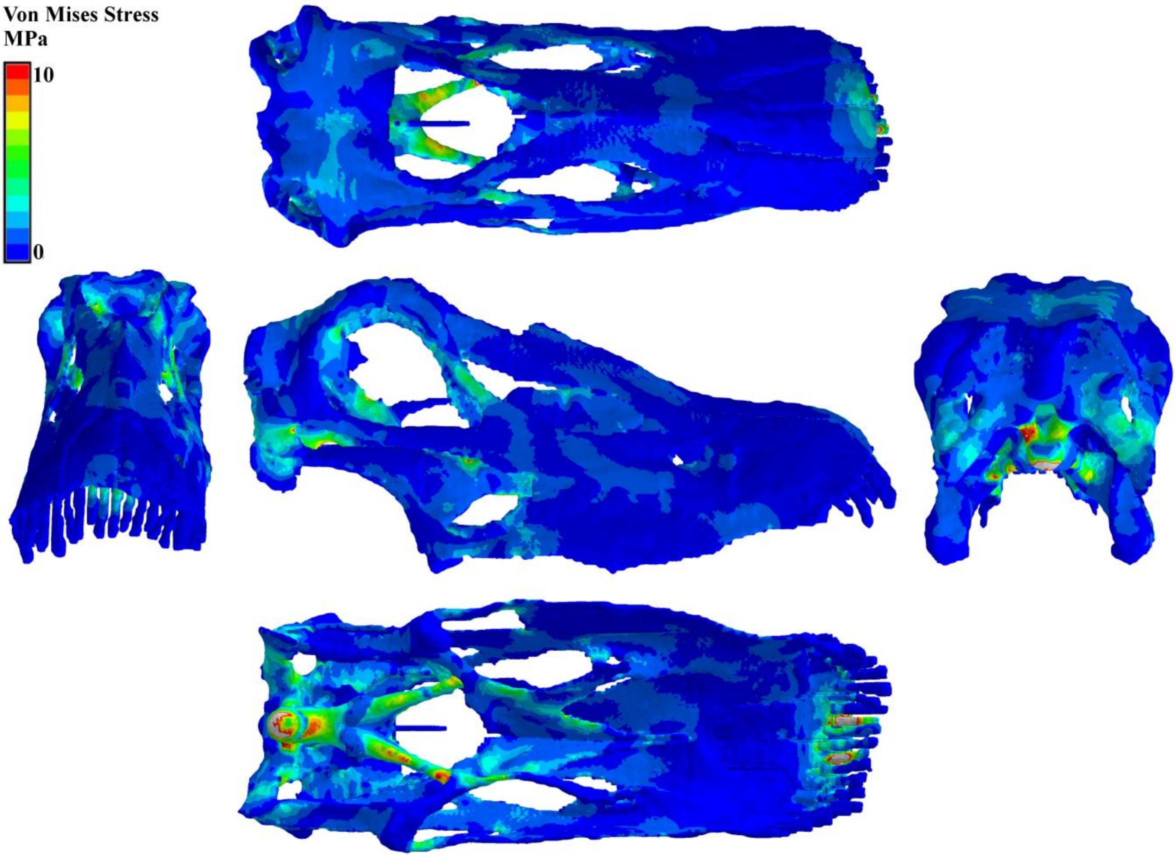


**Figure S23:** FEA results of branch stripping (applied to four teeth) in *Diplodocus.*

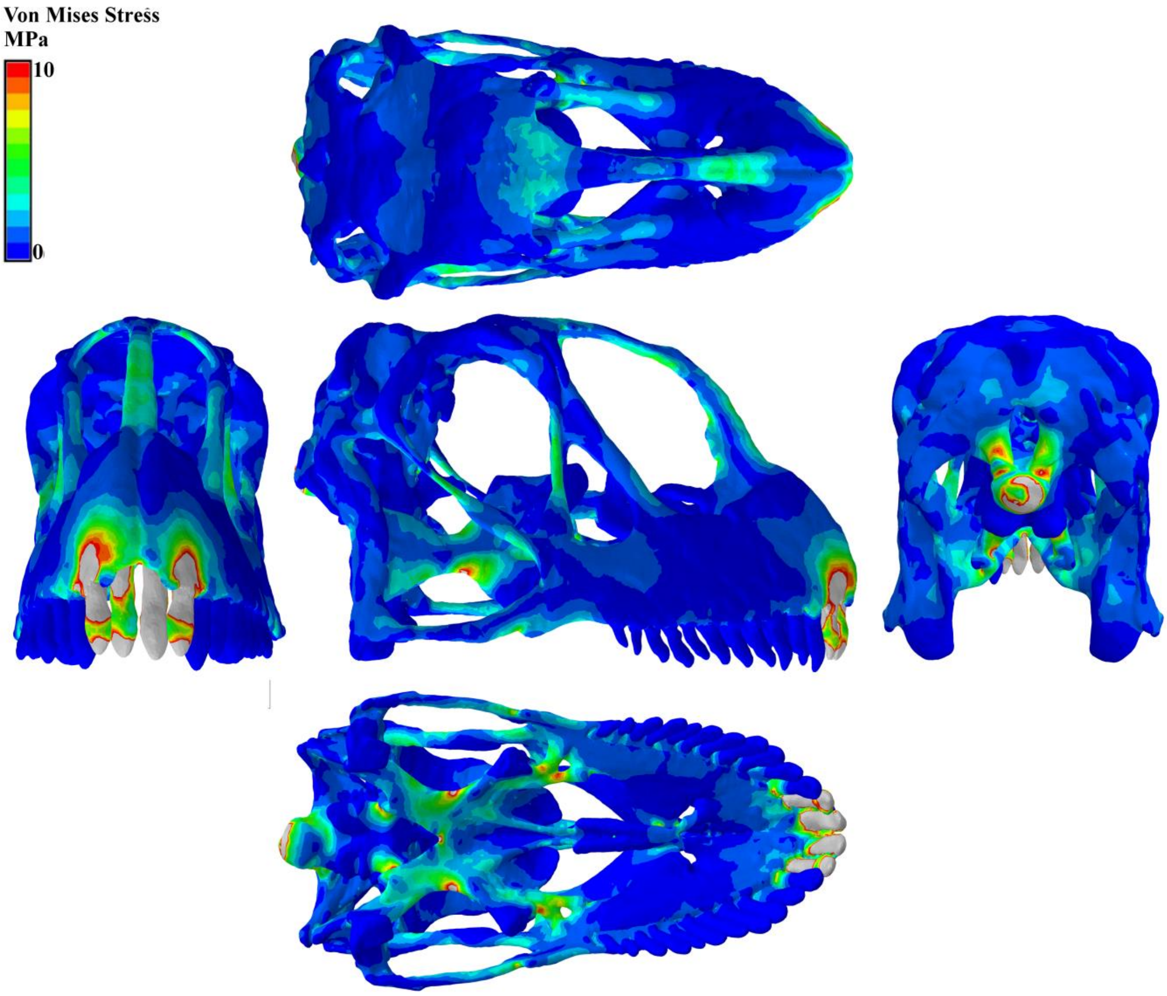


**Figure S24:** FEA results of branch stripping (applied to four teeth) in *Camarasaurus*.

## 12. Supplementary references

[1] Salgado, L., Coria, R. A. & Calvo, J.O. 1997 Evolution of titanosaurid sauropods. I Phylogenetic analysis based on the postcranial evidence. *Ameghiniana* **34**, 3-32.

[2] McPhee, B. W., Yates, A. M., Choiniere, J. N. & Abdala, F. 2014 The complete anatomy and phylogenetic relationships of *Antetonitrus ingenipes* (Sauropodiformes, Dinosauria): implications for the origins of Sauropoda. *Zoological Journal of the Linnean Society*, **171**, 151-205.

[3] D'Emic, M. D. 2013 Revision of the sauropod dinosaurs of the Lower Cretaceous Trinity Group, southern USA, with the description of a new genus. *Journal of Systematic Paleontology*, **11**, 707-726.

[4] Ikejiri, T., Tidwell, V. & Trexler, D. L. 2005 New adult specimens of *Camarasaurus lentus* highlight ontogenetic variation within the species. In *Thunder Lizards: The Sauropodomorph Dinosaurs* (eds. Carpenter, K. & Tidwell, V.) pp. 154-179, Bloomington: University of Indiana Press.

[5] Anderson, P. S. L. 2009 Biomechanics functional patterns, and disparity in Late Devonian arthrodires. *Paleobiology* **35**, 321-342.

[6] Anderson, P. S. L., Friedman, M., Brazeau, M. D. & Rayfield, E.J. 2011 Initial radiation of jaws demonstrated stability despite faunal and environmental change. *Nature* **476**, 206-209.

[7] Anderson, P.S.L., Friedman, M. & Ruta, M. 2013 Late to the table: diversification of tetrapod mandibular biomechanics lagged behind evolution of terrestriality. *Integrative and Comparative Biology* **53**, 283-294.

[8] Stubbs, T. L., Pierce, S. E., Rayfield, E. J. & Anderson, P. S. L. 2013 Morphological and biomechanical disparity of crocodile-line archosaurs following the end-Triassic extinction. *Proc. R. Soc. Lond. B* **280**, 20131940.

[9] Calvo, J. O. 1994 Jaw mechanics in sauropod dinosaurs. *Gaia* **10**, 183-193.

[10] Christiansen, P. 2000 Feeding mechanisms of the sauropod dinosaurs *Brachiosaurus*, *Camarasaurus*, *Diplodocus* and *Dicraeosaurus*. *Historical Biology* **14**, 137-152.

[11] Upchurch, P. & Barrett, P. M. 2000 The evolution of sauropod feeding mechanisms. In *Evolution of Herbivory in Terrestrial Vertebrates: Perspectives from the fossil record* (ed. Sues H-D), pp. 79-122. Cambridge: Cambridge University Press, Cambridge.

[12] Upchurch, P. & Barrett, P. M. 2005 Sauropodomorph diversity through time: macroevolutionary and paleoecological implications. In *The Sauropods: Evolution and Paleobiology* (eds. Curry-Rogers, K. A. & Wilson, J. A.), pp. 104-121. Berkeley: University of California Press.

[13] Wilson, J. A. & Sereno, P. C. 1998 Early evolution and higher-level phylogeny of sauropod dinosaurs. *Society of Vertebrate Paleontology* **18**, 1-79.

[14] Hildebrand, M. 1982 *Analysis of Vertebrate Structure: 2nd Edition*. Hoboken: John Wiley & Sons.

[15] Westneat, M. W. 1994 Transmission of force and velocity in the feeding mechanisms of labrid fishes (Teleostei, Perciformes). *Zoomorphology* **114**, 103-118.

[16] Westneat, M. W. 2003 A biomechanical model for analysis of muscle force, power output and lower jaw motion in fishes. *Journal of Theoretical Biology* **223**, 269-281.

[17] Wainwright, P. C. & Richards, B. A. 1995 Predicting patterns of prey use from morphology with fishes. *Environmental Biology of Fishes* **44**, 97-113.

[18] Sakamoto, M. 2010 Jaw biomechanics and the evolution of biting performance in theropod dinosaurs. *Proceedings of the Royal Society B: Biological Sciences* **277**, 3327-3333.

[19] Stayton, C. T. 2006 Testing hypotheses of convergence with multivariate data: morphological and functional convergence among herbivorous lizards. *Evolution* **60**, 824-841.

[20] Fiorillo, A. R. 1998 Dental microwear patterns of the sauropod dinosaurs *Camarasaurus* and *Diplodocus*: evidence for resource partitioning in the Late Jurassic of North America. *Historical Biology* **13**, 1-16.

[21] Janis, C. M. 1995 Correlations between craniodental morphology and feeding behaviour in ungulates: reciproca illumination betweenliving and fossil taxa. In *Functional Morphology in Vertebrate Paleontology* (ed. Thomason, J. J.), pp. 76-98. Cambridge: Cambridge University Press.

[22] Sues, H.-D. 2000 Herbivory in terrestrial vertebrates: an introduction. In *Evolution of Herbivory in Terrestrial Vertebrates: Perspectives from the Fossil Record* (ed. Sues, H.-D.), pp. 1-9. Cambridge: Cambridge University Press.

[23] Wainwright, S. A., Briggs, W. D., Currey, J. D. & Gosline, J. M. 1976. *Mechanical Design in Organisms*. London: Edward Arnold Publishers.

[24] Vogel, S. 2003 *Comparative Biomechanics: Life's Physical World* Princton: Princeton University Press.

[25] Daegling, D. J. 2001 Biomechanical scaling of the Hominoid mandibular symphysis. *Journal of Morphology* **250**, 12-23.

[26] Summers, A. P., Ketcham, R. A. & Rowe, T. 2004 Structure and function of the Horn Shark (*Heterodontus francisci*) cranium through ontogeny: development of a hard prey specialist. *Journal of Morphology* **260**, 1-12.

[27] Metzger, K. A., Daniel, W. J. T., Ross, C. F. 2005 Comparison of beam theory and finite-element analysis with *in vivo* bone strain data from the alligator cranium. *The Anatomical Record* **283**, 331-348.

[28] Foffa, D., Cuff, A., Sassoon, J., Rayfield, E. J., Mavrogordato, M. & Benton, M. J. 2014. Functional anatomy and feeding biomechanics of a giant Upper Jurassic pliosaur (Reptilia: Sauropterygia) from Weymouth Bay, Dorset, UK. *Journal of Anatomy* **225**, 209-219.

[29] Holliday, C. M. & Nesbitt, S. J. 2013 Morphology and diversity of the mandibular symphysis of archosauriformes. *Geological Society of London, Special Publications* **379**, 555-571.

[30] Porro, L. B., Holliday, C. M., Anapol, F., Ontiveros, L. C., Ontiveros, L. T. & Ross, C. F. 2011 Free body analysis, beam mechanics, and Finite Element Modelling of *Alligator mississippiensis*. *Journal of Morphology* **272**, 910-937.

[31] Walmsley, C.W., Smits, P. D., Quayle, M. R., McCurry, M. R., Richards, H. S., Oldfield, C. C, Wroe, S., Clausen, P. D. & McHenry, C. R. 2013 Why the long face? The mechanics of mandibular symphysis proportions in crocodiles. *PLoS One* **8**, e53873.

[32] Holliday, C. M. 2009 New insights into dinosaur jaw muscle anatomy. *The Anatomical Record* **292**, 1246-1265.

[33] Sereno, P.C., Wilson, J. A., Witmer, L. M., Whitlock, J. A., Maga, A., Ide, O. & Wroe, T. 2007. Structural extremes in a Cretaceous dinosaur. *PLoS One* **2**, e1230.

[34] Boué, C. 1970 Morphologie fonctionnelle des dents labiales chez ruminants. *Mammalia* **34**, 696-711.

[35] Bell, R. H. V. 1971 A grazing ecosystem in the Serengeti. *Sci*. *Am*. **224,** 86–93.

[36] Gordon, I. J. & Illius, A. W. 1989 Resource partitioning by ungulates on the Isle of Rhum. *Oecologia* **79**, 383-389.

[37] Janis, C. M. 1988 Correlation of relative muzzle width and relative incisor width with dietary preference in ungulates. *Zoological Journal of the Linnean Society* **92**, 267-284.

[38] Spencer, L. M. 1995 Morphological correlates of dietary resource partitioning in the African Bovidae. *Journal of Mammalogy*. **76**, 448–471.

[39] Fraser, D. & Theodor, J. M. 2011 Comparing ungulate dietary proxies using discriminant function analysis. *Journal of Morphology* **272**, 1513-1526.

[40] Solounias, N., Teaford, M. & Walker, A. 1988 Interpreting the diet of extinct ruminants: the case of a non-browsing giraffid. *Paleobiology* **14**, 287-300.

[41] Solounais, N. & Moelleken, S. M. C. 1993 Dietary adaptations of some extinct ruminants determined by premaxillary shape. *Journal of Mammalogy* **74,** 1059-1071.

[42] Dompierre, H. & Churcher, C. S. 1996 Premaxillary shape as an indicator of the diet of seven extinct late Cenozoic new world camels. *Journal of Vertebrate Paleontology* **16**, 141-148.

[43] Carrano, M. T., Janis, C. M. & Sepkoski, J. J. Jr. 1999 Hadrosaurs as ungulate parallels: Lost lifestyles and deficient data. *Acta Palaeontologica Polonica* **44,** 237-261.

[44] Whitlock, J. A. 2011 Inferences of Diplodocoid (Sauropoda: Dinosauria) Feeding Behaviour from Snout Shape and Microwear Analyses. *PLoS One* **6**, e18304.

[45] Young, M. T., Rayfield, E. J., Holliday, C. M., Witmer, L. M., Button, D. J., Upchurch, P. & Barrett, P. M. 2012 Cranial biomechanics of *Diplodocus* (Dinosauria, Sauropoda): testing hypotheses of feeding behaviour in an extinct megaherbivore. *Naturwissenschaften* **99**, 637-643. (DOI: 10.1007/s00114-012-0944-y)

[46] Barrett, P. M. & Upchurch, P. 1994 Feeding mechanisms of *Diplodocus*. *Gaia* **10**, 195-204.

[47] Upchurch, P. 1998 The phylogenetic relationships of sauropod dinosaurs. *Zoological Journal of the Linnean Society* **124**, 43-103.

[48] Chure, D., Britt, B. B., Whitlock, J. A. & Wilson, J.A. 2010 First complete sauropod dinosaur skull from the Cretaceous of the Americas and the evolution of sauropod dentition. *Naturwissenschaften* **97**, 379-391.

[49] Barrett, P. M. & Upchurch, P. 2007 The evolution of feeding mechanisms in early sauropodomorph dinosaurs. *Special Papers in Palaeontology* **77**, 91-112.

[50] Allain, R. *et al*. 2004 A basal sauropod dinosaur from the Early Jurassic of Morocco. *Comptes Rendus Palevol* **3**, 199-208.

[51] Allain, R. & Aquesbi, N. 2008 Anatomy and phylogenetic relationships of *Tazoudasaurus naimi* (Dinosauria, Sauropoda) from the late Early Jurassic of Morocco. *Geodiversitas* **30**, 345-424.

[52] Sereno, P. C. & Wilson, J. A. 2005 Structure and function of a sauropod tooth battery. In *The sauropods: evolution and paleobiology* (eds. Curry Rogers, K. A. & Wilson, J. A.), pp. 157-177. Bloomington: University of Indiana Press.

[53] Carballido, J. L., Salgado, L., Pol, D., Canudo, J. I. & Garrido, A. 2012 A new basal rebbachisaurid (Sauropoda, Diplodocoidea) from the Early Cretaceous of the Nequén Basin: evolution and biogeography of the group. *Historical Biology* **24**, 631-654.

[54] Hammer, Ø., Harper, D. A. T. & Ryan, P. D. 2001 Past: Palaeontological statistics software package for education and data analysis. *Palaeontologica Electronica* **4**, 4pp.

[55] Anderson, M. J. 2001 A new method for non-parametric multivariate analysis of variance. *Austral Ecology* **26**, 32-46.

[56] Apaldetti, C., Martinez, R. N., Alcober, O. A. & Pol, D. 2011 A new basal sauropodomorpha (Dinosauria: Saurischia) from Quelbrada del Barro Formation (Marayes-El Carrizal Basin), Northwestern Argentina. *PLoS One* **6**, e26964.

[57] Otero, A. & Pol, D. 2013 Postcranial anatomy and phylogenetic relationships of *Mussaurus patagonicus* (Dinosauria, Sauropodomorpha). *Journal of Vertebrate Paleontology* **33**, 1138-1168.

[58] Wilson, J. A. 2002 Sauropod dinosaur phylogeny: critique and cladistic analysis. *Zoological Journal Linnean Society* **136**, 217-276.

[59] Upchurch, P., Barrett, P. M. & Dodson, P. 2004 Sauropoda. In *The Dinosauria* (eds. Weishampel, D. B., Dodson, P. & Osmólska, H.), pp. 259-322. Berkerly: University of California Press.

[60] Wilson, J. A. & Upchurch, P. 2009 Redescription and reassessment of the phylogenetic affinities of Euhelopus zdanskyi (Dinosauria: Sauropoda) from the Early Cretaceous of China. *Journal of Systematic Palaeontology* **7**, 199-239.

[61] Royo-Torres, R. & Upchurch, P. 2012 The cranial anatomy of the sauropod *Turiasaurus riodevensis* and implications for its phylogenetic relationships. *Journal of Systematic Paleontology* **10**, 553-583.

[62] D'Emic, M. D. 2012 The early evolution of titanosauriform sauropod dinosaurs. *Zoological Journal of the Linnean Society* **166**, 624-671.

[63] Whitlock, J. A. 2011 A phylogenetic analysis of Diplodocoidea (Saurischia: Sauropoda). *Zoological Journal of the Linnean Society* **161**, 872-915.

[64] Fanti, F., Cau, A., Hassine, M. & Contessi, M. 2013 A new sauropod dinosaur from the Early Cretaceous of Tunisia with extreme avian-like pneumatisation. *Nature Communications* **4**, 1-7.

[65] Zaher, H. *et al*. 2011 A complete skull of an Early Cretaceous sauropod and the evolution of advanced titanosaurians. *PLoS One* **6**, e16663.

[66] Mannion, P. D., Upchurch, P., Barnes, R. N. & Máteus, O. 2013 Osteology of the Late Jurassic Portuguese sauropod dinosaur *Lusotitan atalaiensis* (Macronaria) and the evolutionary history of basal titanosauriformes. *Zoological Journal of the Linnean Society* **168**, 98-206.

[67] Klein, N., Sander, P. M., Stein, K., Le Loeuff, J., Carballido, J. L. & Buffetaut, E. 2012 Modified lamellar bone in *Ampelosaurus atacis* and other titanosaurs (Sauropoda): implications for life history and physiology. *PLoS One* **7**, e36907.

[68] Kutty, T. S., Chatterjee, S., Galton, P. M. & Upchurch, P. 2007 Basal sauropodomorphs (Dinosauria: Saurischia) from the Lower Jurassic of India: Their anatomy and relationships. *Journal of Vertebrate Paleontology* **81**, 1218-1240.

[69] Bapst, D. W. 2012 Paleotree: an R package for paleontological and phylogenetic analyses of evolution. *Methods in Ecology and Evolution* **3**, 803-807. (DOI: DOI: 10.1111/j.2041-210X.2012.00223.x)

[70] Brusatte, S. L., Benton, M. J., Ruta, M. & Lloyd, G. T. 2008 Superiority, competition, and opportunism in the evolutionary radiation of dinosaurs. *Science* **321**, 1485-1488.

[71] Revell, L. J. 2012 Phytools: an R package for phylogenetic comparative biology (and other things). *Methods in Ecology and Evolution* **3**, 217-223.

[72] Snively, E. & Russell, A. P. 2007 Functional variation of neck muscles and their relation to feeding style in Tyrannosauroidea and other large theropod dinosaurs. *The Anatomical Record*, **290**, 934-957.

[73] Cleuren, J. & De Vree, F. 2000 Feeding in crocodilians. In *Feeding: Form, Function and Evolution in Tetrapod Vertebrates* (ed. Schwenk K.) pp. 337-358, San Diego: Academic Press.

[74] Tsuihiji, T. 2010. Reconstructions of the axial insertions in the occipital regions of dinosaurs: evaluations of past hypotheses on marginocephalia and tyrannosauridae using the extant phylogenetic bracket approach. *The Anatomical Record* **293**, 1360-1386.

[75] Snively, E., Russell, A. P., Powell, G. L., Theodor, J. M. & Ryan, M. J. 2014. The role of the neck in the feeding behaviour of the Tyrannosauridae: inference based on kinematics and muscle function of extant avians. *Journal of Zoology* **292**, 290-303.

[76] Thomason, J. J. 1991 Cranial strength in relation to estimated biting forces in some mammals. *Canadian Journal of Zoology* **69**, 2326-2333.

[77] Lautenschlager, S. 2012 Cranial myology and bite force performance of *Erlikosaurus andrewsi*: a novel approach for digital muscle reconstructions. *Journal of Anatomy* **222**, 260-272. (DOI: 10.1111/joa.12000)

[78] Madsen, J. H. Jr., McIntosh, J. S. & Berman, D. S. 1995 Skull and atlas-axis complex of the Upper Jurassic sauropod *Camarasaurus* (Reptilia: Saurischia). *Bulletin of Carnegie Museum of Natural History* **31**, 1-115.

[79] Thomason, J. J., Russell, A. P. & Morgeli, M. 1990 Forces of biting, body size, and masticatory muscle tension in the opossum *Didelphis virginiana*. *Canadian Journal of Zoology* **68**, 318–324.

[80] Rayfield, E. J. 2007 Finite Element Analysis and Understanding the Biomechanics and Evolution of Living and Fossil Organisms. *Annual Review of Earth and Planetary Sciences* **35**, 541-576.

[81] Zapata, U. Metzger, K. A., Wang, Q., Dechow, P. C. & Ross, C. F. 2010 Material properties of mandibular cortical bone in the American alligator, *Alligator mississippensis*. *Bone* **46**, 860-867.

[82] Strait, D. S., Wang, Q., Dechow, P. C., Ross, C. F., Richmond, B. G., Spencer, M. A. & Patel, B. A. 2005 Modeling elastic properties in finite element analysis: how much precision is needed to produce an accurate model? *The Anatomical Record* **283**, 275–287.

[83] Bright, J. A. & Rayfield, E. J. 2011 Sensitivity and *ex vivo* validation of finite element models of the domestic pig cranium. *Journal of Anatomy* **219**, 456-471.

[84] Curry, K. A. 1999 Ontogenetic histology of *Apatosaurus* (Dinosauria: Sauropoda): New insights on growth rates and longevity. *Journal of Vertebrate Paleontology* **19**, 654-665.

[85] Reilly, D., Burstein, A. 1975 The elastic and ultimate properties of compact bone tissue. *Journal of Biomechanics* **8**, 393–405.

[86] Gilmore, R. S., Pollack, R. P. & Katz, J. L. 1969 Elastic properties of bovine dentine and enamel. *Archives of Oral Biology* **15**, 787–796.

[87] Ichim, I., Schmidlin, P. R., Kieser, J. A. & Swain, M. V. 2007 Mechanical evaluation of cervical glass-ionomer restorations: 3D finite element study. *Journal of Dentistry* **35**, 28–35.
